# Differences between surfactant-free Au@Ag and CTAB-stabilized Au@Ag star-like nanoparticles in the preparation of nanoarrays to improve their surface-enhanced Raman scattering (SERS) performance†

**DOI:** 10.1039/d3na00483j

**Published:** 2023-09-04

**Authors:** Sy Van Vu, Anh-Thu Nguyen, Anh-Thi Cao Tran, Viet-Ha Thi Le, Tien Nu Hoang Lo, Thi H. Ho, Nguyet. N. T. Pham, In Park, Khuong Quoc Vo

**Affiliations:** a Faculty of Chemistry, University of Science, Vietnam National University – Ho Chi Minh City 227 Nguyen Van Cu Street, Ward 4, District 5 Ho Chi Minh City 70000 Vietnam vqkhuong@hcmus.edu.vn; b Vietnam National University Ho Chi Minh City Vietnam; c Research Institute of Clean Manufacturing System, Korea Institute of Industrial Technology (KITECH) 89 Yangdaegiro-gil, Ipjang-myeon Cheonan 31056 South Korea; d KITECH School, University of Science and Technology (UST) 176 Gajeong-dong, Yuseong-gu Daejeon 34113 South Korea; e Laboratory for Computational Physics, Institute for Computational Science and Artificial Intelligence, Van Lang University Ho Chi Minh City Vietnam; f Faculty of Mechanical – Electrical and Computer Engineering, School of Technology, Van Lang University Ho Chi Minh City Vietnam

## Abstract

In this study, we assessed the controlled synthesis and efficacy of surface-enhanced Raman scattering (SERS) on two distinct types of star-like Au@Ag core–shell nanoarrays. These nanoarrays were designed based on gold nanostars (AuNSs), which were synthesized with and without CTAB surfactant (AuNSs-CTAB and AuNSs-FS, respectively). The AuNS-FS nanoparticles were synthesized *via* a novel modification process, which helped overcome the previous limitations in the free-surfactant preparation of AuNSs by significantly increasing the number of branches, increasing the sharpness of the branches and minimizing the adsorption of the surfactant on the surface of AuNSs. Furthermore, the differences in the size and morphology of these AuNSs in the created nanoarrays were studied. To create the nanoarrays, a three-step method was employed, which involved the controlled synthesis of gold nanostars, covering them with a silver layer (AuNSs-FS@Ag and AuNSs-CTAB@Ag), and finally self-assembling the AuNS@Ag core-shelled nanoparticles *via* the liquid/liquid self-assembly method. AuNSs-FS@Ag showed higher ability in forming self-assembled nanoarrays than the nanoparticles prepared using CTAB, which can be attributed to the decrease in the repulsion between the nanoparticles at the interface. The nano-substrates developed with AuNSs-FS@Ag possessed numerous “hot spots” on their surface, resulting in a highly effective SERS performance. AuNSs-FS featured a significantly higher number of sharp branches than AuNSs-CTAB, making it the better choice for creating nanoarrays. It is worth mentioning that AuNSs-CTAB did not exhibit the same benefits as AuNSs-FS. The morphology of AuNSs with numerous branches was formed by controlling the seed boiling temperature and adding a specific amount of silver ions. To compare the SERS activity between the as-prepared nano-substrates, *i.e.*, AuNS-CTAB@Ag and AuNS-FS@Ag self-assembled nanoarrays, low concentrations of crystal violet aqueous solution were characterized. The results showed that the developed AuNSs-FS@Ag could detect CV at trace concentrations ranging from 1.0 ng mL^−1^ to 10 ng mL^−1^ with a limit of detection (LOD) of 0.45 ng mL^−1^ and limit of quantification (LOQ) of 1.38 ng mL^−1^. The nano-substrates remained stable for 42 days with a decrease in the intensity of the characteristic Raman peaks of CV by less than 7.0% after storage. Furthermore, the spiking method could detect trace amounts of CV in natural water from the Dong Nai River with concentrations as low as 1 to 100 ng mL^−1^, with an LOD of 6.07 ng mL^−1^ and LOQ of 18.4 ng mL^−1^. This method also displayed good reproducibility with an RSD value of 5.71%. To better understand the impact of CTAB stabilization of the Au@Ag star-like nanoparticles on their surface-enhanced Raman scattering (SERS) performance, we conducted density functional theory (DFT) calculations. Our research showed that the preparation of AuNSs-FS@Ag *via* self-assembly is an efficient, simple, and fast process, which can be easily performed in any laboratory. Furthermore, the research and development results presented herein on nanoarrays have potential application in analyzing and determining trace amounts of organic compounds in textile dyeing wastewater.

## Abstract 2

Illustrating the differences in the structure and morphology of Au@Ag star-like nanoparticles between synthesized with and without surfactant in developing SERS substrates.

## Graphical abstract

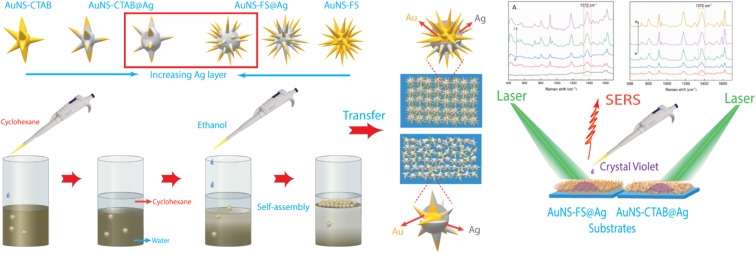

## 1. Introduction

Recently, surface-enhanced Raman scattering (SERS) has emerged as one of the most promising techniques for many applications including biosensors,^1–3^ materials science,^4^ analytical chemistry,^5,6^ food chemistry,^7^ bioanalysis and diagnosis.^8^ The SERS signal enhancement strongly depends on the interaction between the induced light and substrate carrying the analyte.^9–11^ Based on the electromagnetic enhancement mechanism,^12^ SERS signal amplification relies on the intersection of plasmonic nanoparticles, which creates a strong near-field coupling effect called “hot spots”.^13^ The “hot spots” are the positions where the localized electromagnetic field can be highly improved, and the Raman signal can be significantly amplified when the analytes reaches these positions. Thus, SERS substrates have been designed to achieve as many “hot spots” as possible to maximize the Raman signal enhancement. In recent years, the interest in developing SERS substrates has tremendously increased, especially in refining the intrinsic shape of metal nanoparticles. Noble metallic nanoparticles such as silver and gold are often the preferred materials to develop various types of SERS substrates due to their rich plasmonic resonance properties in the near-infrared (NIR) to visible range. In particular, gold nanostars (AuNSs) are superior the other anisotropic nanoparticles due to their advantages of inert, stable, easily tailored synthesis and many geometrical shapes.^14,15^ They satisfy the above-mentioned requirements because of their specific tunable plasmonic properties ranging from the visible to near-infrared region, which resonate with the exciting light source. Besides, gold nanostars can provide high surface activity due to the small size effect, and their sharp branches can create more intersection spaces between particles.^9,16^ The symmetry and high aspect ratio of branches on AuNSs also determine their SERS enhancement ability.^17,18^ Gold nanostars synthesized without surfactant or capping agent can provide many advantages in optical analysis methods.^19^ The original procedure for the surfactant-free synthesis of AuNSs proposed by Vo-Dinh and coworkers with the addition of ascorbic acid and AgNO_3_ can produce a high yield of gold nanostars.^19^ However, this approach can still be improved in terms of the homogeneity of the particle size and a larger number of AuNS branches can be generated. Subsequently, Indrasekara *et al.* presented an optimized bottom-up synthesis approach to improve the reproducibility and homogeneity of AuNSs to reliably obtain the desired optical properties. In the proposed synthesis approach, the synthesis parameters must be tightly controlled, such as the concentration of reactive Au to Ag and Au to Au seeds.^20^ Although this approach can produce more homogeneous AuNSs than the previous method, the number of the branches still did not clearly increase (about 10–15 branches), and the length of the branches formed upon close observation of the TEM images are not equal.^20^ Ziwei Ye *et al.* reported a new method for the surfactant-free synthesis of spiky hollow Ag–Au nanostars with chemically exposed surfaces for developing SERS single particles with hydroxyethyl cellulose as a weakly bound stabilizing agent. Each spiky gold nanoparticle consisted of more than 50 spikes on their surface and a hollow cubic core with a diameter of 150 nm.^21^ The synthesis process was directly conducted with the precursors, reducing agent, and addition of a weakly bound protecting agent to form stable colloids. However, this direct approach can result in the formation of by-products such as spherical-shape nanoparticles. Thus, the authors performed a centrifugation step to purify the resulting nanoparticles to retain the spiky nanoparticles and remove the unwanted by-products.^21^ To overcome the above-mentioned limitations, we proposed a novel modification method, which is more straightforward to achieve the controlled synthesis of AuNSs, by maintaining the seed synthesis step and the growth process at an appropriate temperature and combining different amounts of ascorbic acid and AgNO_3_ to produce high-aspect ratio AuNSs and increase the number of branches (>50 spikes) protruding from their core.

In the last few decades, to increase the SERS enhancement ability of gold nanoparticles, a new class of core–shell nanostructures has been intensively studied based on their specific optical characteristics.^22–26^ The properties of these novel nanomaterials are different and better compared to that of their individual materials. Typically, AuNPs are widely known for their inert nature and biocompatibility but they only exhibit average SERS enhancement.^27^ Alternatively, silver nanostructures possess an excellent enhanced Raman signal,^28^ but they are easily oxidized or unsuitable for *in vivo* application. Therefore, a finely deposited silver layer on the core of gold nanoparticles can help avoid the loss of their SERS activities. Various Au–Ag core–shell nanomaterials have been prepared to improve their SERS performance using strategies such as the synthesis of core–shell Au@Ag nanoparticles proposed by Li *et al.* using the microfluidic technique.^29^ Fan *et al.* prepared Au–Ag bimetallic nanoparticles with diameters of approximately 3–5 nm and examined their SERS properties with different reporter molecules.^30^ Furthermore, if star-like nanoparticles are covered with a silver shell, and then orientally self-assemble into an array structure, they can deliver a greatly intensified Raman signal due to the utilization of not only the advantages of Au and Ag nanoparticles but also the creation of large void areas and nanogaps. Two-dimensional Au@Ag core–shell nanocubes were fabricated by Dong *et al.* for ultrasensitive SERS detection.^31^ Although the above-mentioned studies on core–shell nanostructures have great potential for application in developing SERS substrates with ultrasensitive ability, the fabrication process needs to be simplified and requires some specialized equipment.

The assembly of nanoparticles (NPs) into well-ordered structures shows excellent promise as nanomaterials for high-performance SERS due to the strong electromagnetic field generated by the ultrasmall gaps between the adjacent nanoparticles.^13,32^ In this case, various techniques have been proposed to arrange nanoparticles in the arrays to fabricate SERS substrates, such as slow drying by radiative heating,^33^ lithography,^34^ self-assembly of nanoarrays using modified ligands,^35^ layer-by-layer assembly,^36,37^ liquid/liquid interface self-assembly (LLISA),^38^ use of multi-dentate ligands,^39^ and template-mediated assembly. Among them, liquid/liquid interface self-assembly (LLISA) is a facile,^40^ fast, and low-cost technique to prepare high-ordered nanoarrays.^41,42^ The interface between oil and aqueous liquids offers a vital region for self-assembling particles at the nanoscale level, resulting in the formation of membrane-like structures. Many of the properties of these membrane-like structures are more advanced than their individual nanoparticles. Moreover, the membrane structure fabricated by LLISA could help to purify and separate nanoparticles with selected sizes for developing sensors and catalysts.^43^

In recent years, the self-assembly process has been researched on various types of noble metallic nanoparticles for maneuvering novel SERS-active substrates. Pu *et al.* synthesized a sensitive SERS substrate based on an Au@Ag core–shell nanostar nanoarray to detect thiram in apples at the detection limit of 0.018 mg L^−1^.^44^ Mao *et al.* presented a large-scale monolayer at the cyclohexane/water interface for designing SERS active substrates to determine methamphetamine at 100 ppb using a portable Raman spectrometer. Polyvinyl pyrrolidone (PVP) was used to exchange cetyltrimethylammonium bromide (CTAB) stabilized on the surface of nanostars.^45^ However, preparing large-dimension ordered star-like nanoparticles covered with a silver shell and attaining a self-assembly interface layer are still considerable challenges. However, these methods have some limitations, such as low efficiency of the formation of nanoarray structures, nanoparticles do not form a uniform layer, and poor SERS signal amplification. The main disadvantage of creating a nanoarray structure at the interface between two phases using anisotropic nanoparticles is the bulk quantities of precursors required for the synthesis of gold nanoparticles.^46^ Furthermore, excess surfactant induces the dispersion effect of nanoparticles in the aqueous phase, thus hindering the migration of the anisotropic nanoparticles to the interface. Chunchun Li *et al.* discovered the concentration threshold of CTAB for the creation of AuNS arrays. The authors reported that a metal-liquid-like layer would form once citrate-reduced gold colloids were mixed with an appropriate amount of CTAB in the range of 5 × 10^−6^ to 8 × 10^−5^ M and the dichloromethane phase.^46^ However, in the experiment, the influence of CTAB at this minimal concentration threshold is quite challenging, especially for the synthesis of AuNSs with an anisotropic morphology because the seed-mediated method requires a large quantity of CTAB. In the case of CTAB removal treatment, it is challenging to completely remove the CTAB on the surface of the nanoparticles. Therefore, the development of nanoarrays based on surfactant-free nanoparticles is necessary, which can limit the disadvantages and limitations associated with the use of CTAB.

Crystal violet (CV) is a dye with a positive charge, which is commonly used to stain biological specimens, dye cotton and wool, color hair, and various textile processes.^47,48^ It is a type of colored organic compound that contains triphenylmethane structures. CV, which has a blue-violet appearance, is formally known as tris(4-(dimethylamino)phenyl)methylium chloride according to IUPAC nomenclature. It has been found to be unreactive and incompatible with strong oxidizing agents and acids.^49^ Exposure to CV can cause moderate eye irritation and sensitivity to light, potentially leading to permanent damage to the cornea and conjunctiva.^50^ Also, it is highly toxic to human and mammalian cells, causing irritation in the skin and digestive tract.^51^ In extreme cases, it can have severe effects on the kidneys^53^ and respiratory system.^52^ Therefore, it is crucial to measure the amount of CV present in natural water samples to quickly manage and reduce the release of industrial waste into the environment, thus minimizing any potential harm to human health.

Herein, we present a simple and effective method for the preparation of SERS substrates using a nanoarray structure made of gold nanostars coated with a silver layer (AuNSs@Ag). This method is highly effective for the creation of SERS substrates with exceptional enhancement efficiency. To examine and assess the variation in performance between two distinct types of gold nanostars (AuNSs) in the fabrication process of the nanoarray structure, they were prepared using different techniques, *i.e.*, using CTAB as the structure-directing agent (AuNSs-CTAB) and without surfactant (AuNS-FS) *via* a seed-mediated process. In the case of the AuNSs synthesized without surfactant, the synthetic process was modified to achieve a different nanostar morphology from the previous studies with high numbers of branches, which is beneficial for developing SERS substrates. The exceptional morphology with a high aspect ratio of branches was simply governed by controlling the temperature for the preparation of the seed colloids and the AuNS growth procedure, AgNO_3_ concentration, amount of seed colloids, and ascorbic acid concentration.

Subsequently, nanoarrays were fabricated using the two types of as-synthesized nanoparticles based on the cyclohexane/water interface self-assembly method, and membrane-like structures were spontaneously formed after the dropwise addition of ethanol into the two immiscible liquids. The AuNSs prepared without CTAB showed a higher ability to self-assemble at the interface of the cyclohexane/water phases than the that synthesized with CTAB, as examined with the microscopy technique. This can be ascribed to the reduced repulsion between the nanoparticles, which was due to the presence of surfactant molecules on the surface of the AuNS nanoparticles in the case of synthesized with CTAB.

**Fig. 1:**
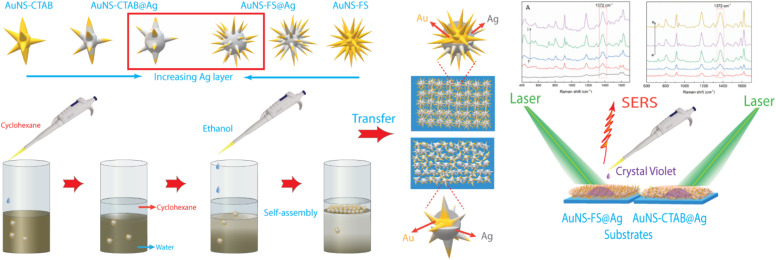
Scheme of the controlled synthesis of gold nanostar with CTAB and without the use of surfactant molecules, illustrating the differences in the structure and morphology of star-like nanoparticles. The as-synthesized AuNSs-CTAB and AuNSs-FS were shelled by a silver layer, self-assembled using the liquid/liquid interface self-assembly technique, and employed in developing SERS nano-substrates.

The nanoarrays were placed on a glass slide to create an SERS substrate and left to dry at room temperature in the open air. In Raman the experiments, it was found that the nanoarrays made with AuNSs-FS@Ag were more effective in enhancing the detection of crystal violet (CV) than those made with AuNSs-CTAB@Ag. The enhancement efficiency was particularly high at low concentrations of 1.0 and 10 ng mL^−1^. Furthermore, we also used the AuNS-FS@Ag nanoarrays to identify trace amounts of CV in natural water samples taken from the Dong Nai River in Dong Nai Province. We also performed density functional theory (DFT) calculations to study the role of CTAB stabilization of the Au@Ag star-like nanoparticles in their surface-enhanced Raman scattering (SERS) performance (Fig. 1).

## 2. Results and discussion

### 2.1. Optical and morphological properties

**Fig. 2:**
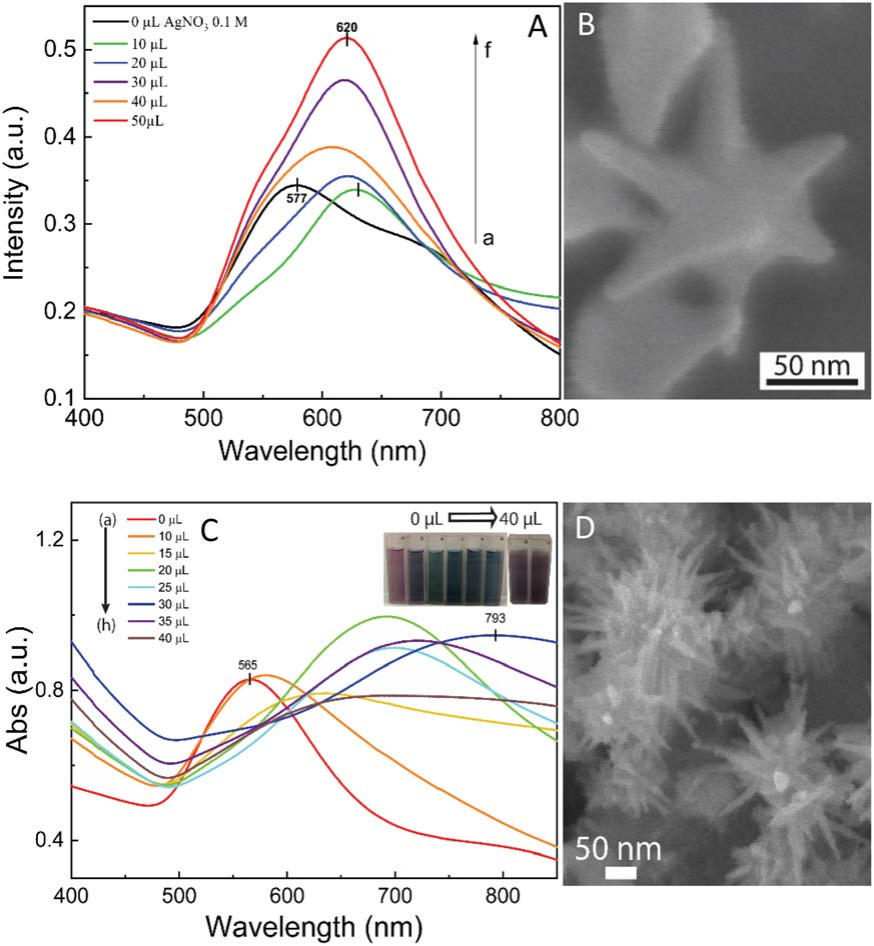
(A) UV-Vis spectrum of the as-prepared AuNS-CTAB colloidal samples shows a blue shift from 630 and 628 to 620 nm with an increasing volume of 0.1 M AgNO_3_ from 0 to 50 μL, respectively. (B) Corresponding SEM micrograph of fine-tuned AuNS particles with eight symmetrical spikes formed using 10 μL of 0.1 M AgNO_3_, 0.1 M ascorbic acid, and 0.1 M CTAB. (C) UV-Vis absorbance spectrum of AuNS-FS colloids presents broad bands above 700 nm, indicating the presence of a spike morphology. With an increase in the volume of 0.01 M AgNO_3_, the spectrum showed a redshift in these bands. (D) SEM morphology of AuNSs synthesized without using surfactant with many long sharp spikes with a small core at 30 μL of 0.01 M AgNO_3_, 10 μL of 0.1 M HCl, and 100 μL of seed colloid.

The self-assembly of nanoparticles at the water/oil interface depends on many factors, including the size, morphology, and surface charge of the particles, and ratio of the water/oil phase. Thus, the controlled synthesis of AuNSs with a specific morphology was the focus to achieve high SERS substrate repeatability and sensitivity. Considering the above-mentioned factors, manipulation of the AgNO_3_ additive amount was required to control the number, symmetry, and relative spike lengths of AuNSs. A series of experiments was conducted with an increasing volume of 0.1 M AgNO_3_ from 0 and 10 to 50 μL (Fig. 2). Simultaneously, the other factors, such as ascorbic acid, seed, and growth solution, were kept constant at 0.1 M, 0.8 mL, and 10 mL, respectively.

**Fig. 3:**
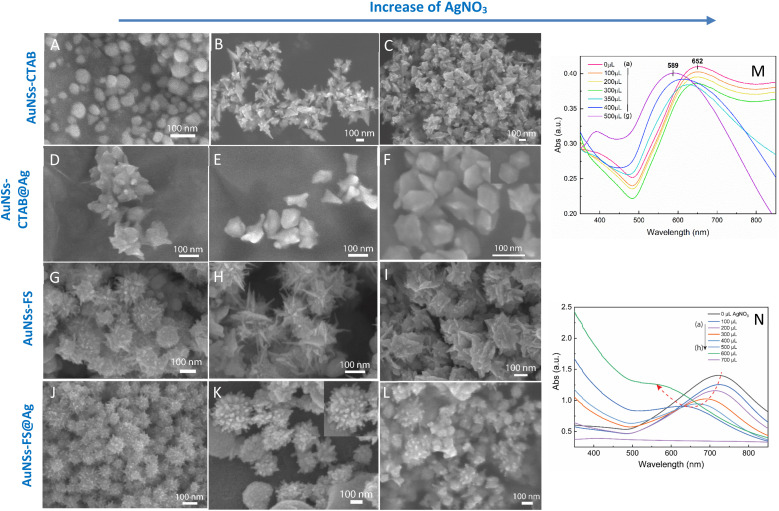
SEM micrographs of (A–C) AuNSs-CTAB synthesized with of 0.8 mL of seed solution and 10 mL of growth solution containing 1.0 mL of 25 mM HAuCl_4_, 0.1 M CTAB, and 0.05 mM AgNO_3_ with different volumes of (A) 0, (B) 20, and (C) 30 μL (scale bars for all are 100 nm). (D–F) AuNSs coverage with Ag layers with a variation in the volume of AgNO_3_ of (D) 100, (E) 200, and (F) 300 μL of 10 mM AgNO_3_, with the other conditions of 275 μL of 10 mM ascorbic acid and 5 mL of AuNS colloidal solutions kept constant. (G–I) SEM images of gold nanostars with an increase in sharp spikes synthesized *via* the seed growth route without using surfactant for each additional volume of (G) 10, (H) 20, (I) 30 μL of 0.01 mM AgNO_3_, while the remaining factors including 10 mL of 0.25 mM HAuCl_4_, 10 μL of HCl 1.0 M, and 100 μL of the seed solution were unchanged. (J–M) Morphologies of AuNSs changed after covering with an Ag^0^ layer, where the tips became blunted and the core star grew bigger at (J) 100, (K) 200, and (L) 300 μL of 10 mM AgNO_3_. Corresponding UV-Vis spectra of (M) AuNSs-CTAB@Ag prepared at various 10 mM AgNO_3_ volumes ranging from 0 to 500 μL and (N) AuNSs-FS@Ag at different volumes of 10 mM AgNO_3_ from 0 to 700 μL.

The UV-Vis spectra of the colloid samples show the surface plasmon resonance peaks at around 620–630 nm, suggesting the formation of star-like particles,^53^ and the narrow plasmon band indicates the formation of AuNSs-CTAB with a few spikes in the colloidal solution (Fig. 2A). In addition, the growth of the spike could induce a red-shift in the characteristic local surface plasmon resonance (LSPR) peak in the wavelength range of 577 to 630 nm.^54^ Notably, the seed-mediated protocol based on CTAB can produce asymmetric and homogeneous structures with well-defined spikes.^55^ The scanning electron microscopy (SEM) results also support this point. The representative SEM micrograph in Fig. 2B shows that the typical star-like particle contained eight symmetrical spikes, which grew from the core to various directions, and the lengths of these spikes were almost equal. The combination of CTAB and AgNO_3_ could improve the blocking of specific crystallographic facets, which facilitated the adsorption of Au^0^ in the other planes to yield particles with an asymmetric multi-spiked morphology.^55,56^Fig. 3B and C show that the AuNSs were well-formed with a size of approximately 150–170 nm and core diameter of 72 nm. The particles were formed with a minimum of four to six branches elongated in certain directions with different lengths. The appropriate adjustment of the AgNO_3_ volume (20 μL, 0.1 M) resulted in the production of a large amount of AuNSs-CTAB in aqueous solution with symmetrically shaped and well-formed spikes (Fig. 3B), confirming with the plasmonic peaks that appeared at around 628 nm in the UV-Vis spectrum (Fig. 2A). When a larger quantity of Ag^+^ was used in the reaction mixture, corresponding to 30 μL, the star-shaped particles were re-shaped with thicker and shorter spikes, which are associated with the blue shift in the SPR peaks (Fig. 3C and 2A, curve d). The average size of AuNSs (approximately 150 nm), measured using the distance between two opposite spike tips, significantly decreased (∼20 nm) after the addition of more Ag^+^ ions (30 μL of 0.1 M AgNO_3_). When more than 30 mL of AgNO_3_ was used, the spikes did not elongate as expected. This was because some of the Ag^+^ was partially adsorbed on the tips. Alternatively, in the absence of Ag^+^ ions in the reaction system, most of the formed nanoparticles were pseudo-spherical, and almost no star-liked particles were detected in the SEM images (Fig. 3A). Furthermore, the UV-Vis spectrum of the sample synthesized in the absence of AgNO_3_ also showed apparent differences compared to the other samples, which exhibited an SPR peak located at 577 nm (Fig. 2A, curve black).

In the case of the gold nanostars synthesized *via* the surfactant-free approach during the seed preparation process, TSC did not act as a protecting agent but helped stabilize the seeds by binding to their surface. This resulted in an overall negative charge on the seed surface, which prevented the agglomeration of the particles.^57^ TSC was utilized as a reducing agent to form gold seed nanoparticles, which acted as nuclei for the selective deposition of Au atoms. To obtain the desired seed particles for growing multi-branched gold nanostars, the seed preparation should be done at high temperatures given that TSC is not an effective reducing agent at room temperature. To determine the appropriate conditions, the boiling temperature of the HAuCl_4_ precursor solution was examined in the range of 40 °C to 100 °C before the addition of TSC. By analyzing the UV-Vis spectra of the seed solution synthesized at varying reaction temperatures (Fig. S2†), that the presence of a resonance peak in the range of 530–590 nm was observed, indicating the formation of gold nanoparticles.^58^ As the temperature increased from 40 °C to 60 °C, a plasmon band was observed near 541 nm, which then shifted to 551 nm and 554 nm with a corresponding increase in absorbance intensity (Fig. S2,† curve e and f). This suggests that at higher temperatures, more TSC can reduce Au^3+^ to Au^0^ atoms, resulting in the formation of smaller nanoparticles. At a temperature of 70 °C, there was a noticeable shift in the plasmon towards a longer wavelength of 594 nm (Fig. S2,† curve d), but the absorption intensity decreased. This can be attributed to the agglomeration of the seeds, leading to a reduction in the number of seeds at the nanometer level.

By increasing the temperature of the gold precursor to 90 °C, there was a noticeable decrease in the absorption peak at 594 nm (Fig. S2,† curve b). Additionally, the plasmon band shifted towards a shorter wavelength of around 550 nm. This change can be attributed to the fact that at higher temperatures, HAuCl_4_ transforms into more [AuCl_3−*x*_(OH)_*x*+1_], leading to fewer initial [AuCl_4_^−^], and consequently the formation of fewer seed.^59^ According to the TEM study, nanoparticles with a size of approximately 20 nm were formed when the synthesis was performed at a temperature of 60 °C (Fig. S2B†). However, when the temperature was increased to 90 °C, the nanoparticles started to self-aggregate (Fig. S2C†). These TEM findings are consistent with the predictions based on the UV-Vis spectral observations. Therefore, a heating temperature of around 60 °C was considered appropriate for further investigation.

We also visually evaluated the absorbance spectrum of the AuNS colloids prepared through the surfactant-free approach by regulating the volume of 0.01 M AgNO_3_ (Fig. 2C). The plasmonic band shape gradually changed from that observed for the AuNS-CTAB colloids. These bands were relatively broad and became red-shifted with an increase in the amount of AgNO_3_ (Fig. 2C, curve a–h), indicating that a different type of star-like morphology was formed. According to the SEM images presented in Fig. 2D, this type of nanostar has numerous sharper spikes and smaller core sizes. SEM micrographs of the AuNS samples synthesized with various volumes of 0.01 M AgNO_3_ (10, 20, and 30 μL) were also taken to evaluate the effect of quantity of Ag^+^ ions the on the morphology of AuNSs, as presented in Fig. 3G and I. Notably, the AuNSs at 10 μL of 0.01 M AgNO_3_ were comprised of many protrusions with short tips (Fig. 3G), while the particles formed at 20 μL had thinner spikes with sharper edges (Fig. 3H). With an increase in the concentration of AgNO_3_ (30 μL of 0.01 M Ag^+^), AuNSs appeared with fewer spikes, and the length of the spikes was shorter than that of the particles formed at 20 μL (Fig. 3I). Therefore, the shape of the spikes could be modulated by changing the Ag^+^ concentration while keeping a certain amount of seed. The role of the Ag^0^ atom was previously proposed to be the preferential adsorption site on specific facets of the seed particles.^60^ Due to the underpotential deposition phenomenon,^61^ the arrangement of silver atoms and their closest Au neighbors varies depending on the fcc crystalline facets of the gold nanoparticles when Ag^0^ is deposited on AuNPs. On the Au (111) facets, three of the closest Au neighbors surround each silver atom. On the Au (100) surfaces, each silver atom has four nearest Au atoms, while on the Au (110) surfaces, each silver atom has five nearest Au atoms, with one located in the second layer below the silver atom. Thus, more Ag^0^ atoms tend to accumulate on the (110) facet compared to the (100) and (111) facets. This can limit the surface area of the (110) facet and promote the anisotropic growth of the gold nanoparticles.^62,63^ Consequently, Au^0^ atoms will naturally deposit on the energetically favorable (100) and (111) facets to form star-like nanoparticles. The effect of ascorbic acid and HAuCl_4_ on the formation of AuNSs-FS was also studied to find the appropriate conditions for the controlled synthesis of AuNSs (Fig. S3 and S4,† respectively, in the ESI†). AuNSs-FS were further coated with an Ag layer to utilize the SERS enhancement abilities of Ag and Au when excited by incident light with an appropriate wavelength.^64^

The sharp tips of the Au nanostars (AuNSs) significantly enhanced the electromagnetic field surrounding the sharp spikes, which led to an increase in the population of hot electrons near the interface,^65^ resulting in an improvement in the optical efficiency in both the visible and NIR regions compared to spherical or rod-shaped Au nanostructures. Thus, the intense field enhancement at the sharp tips of nanostars makes this shape an excellent candidate for SERS applications.^66^ Moreover, when Au nanostars were coated with a thin layer of Ag, they enhanced the plasmonic properties of the Ag layer and the chemical stability of Au combined in the single bimetallic Au/Ag core–shell nanostructure, together with the appearance of a tunable LSPR band in the visible to NIR region by simply changing the thickness of the Ag coating over the Au nanostars.^67^ Both the optical and catalytic properties of these nanostructures can be tuned by controlling their morphology (such as sharpness and number of tips surrounding the core) and varying the shell thickness of the nanostructures. These design-controlled nanomaterials enable the potential of plasmonic catalysis for a fundamental understanding of the underlying mechanism, as well as optimization of their efficiency. Gagandeep Kaur *et al.*^68^ synthesized Au NS covered with a thin Ag layer with multiple sharp tips, which exhibited intense LSPR and very strong electric fields were created at the tips. The enhancement in the electric field obtained from the Au core/Ag shell NS compared to pure Au NS resulted in a higher SERS enhancement for the AuNSs core/Ag shell than that of the pristine AuNSs. Therefore, in this study, we coated AuNSs with a thin Ag layer and further investigated the SERS structure.

### 2.2. Covering AuNSs-CTAB and AuNSs-FS with an Ag layer

The process for the coverage of the AuNS cores with a silver layer was also monitored through UV-Vis spectroscopy to distinguish between the formation of the silver layer on the surface of AuNSs and the formation of separate silver nanoparticles in the colloidal solution. To investigate the influence of the AuNS core morphologies and their optical properties on the improvement in SERS, the AuNSs-CTAB with the SPR peak at 650 nm and AuNSs synthesized without using surfactant with a plasmonic peak at 730 nm were chosen to be covered with Ag. In the case of the coverage of the Ag layer on the Au core, it is challenging to distinguish the plasmonic band of the silver and gold nanoparticles. However, if the silver nanoparticles exist independently besides AuNSs, two separate peaks would arise in the spectrum of the colloid samples.^69^ In the case of the silver covering on AuNSs stabilized with CTAB, a similar process was conducted as previous works^6,70^ with some modification. We partially removed the unadsorbed CTAB in solution before covering, and the AuNSs were selected based on the appropriate condition that produced the nanoparticles with long spike lengths and highly symmetric spikes (for further information on the washing process involving the CTAB surfactant, please see the Experimental section in the ESI†). The morphologies of AuNSs before and after removing the unadsorbed CTAB in the solution were further investigated through TEM and HRTEM (Fig. S6A and B,† respectively). Almost no transformation in the particle shape was observed after removing CTAB, while the spike length was not shortened and its sharpness was retained (Fig. S6B†).

As shown in Fig. 3M, the SPR peak appeared at 652 nm for the AuNS-CTAB@Ag sample, which was blue-shifted to 605 nm when the volume of AgNO_3_ used in the AuNS core coverage increased from 0 to 350 μL (Fig. 3M, curve a–e). This result indicates the coverage of a silver layer on the AuNS core.^71^ However, the use of too much Ag^+^ ions (500 μL) led to the formation of two separate peaks at around 589 and 390 nm (Fig. 3M, curve g), implying the appearance of independent silver nanoparticles beside the AuNSs in the reaction mixture. Similar results were previously reported by Zhu *et al.*^70^ The changes in the star-like particle morphology after covering with Ag were also studied. Fig. 3D–F show the representative SEM images of AuNSs-CTAB covered with a silver layer at 100, 200, and 300 μL of 10 mM AgNO_3_. It was found that the AuNS-CTAB particles transformed from a spinous to polygonal shape with the increasing addition of Ag^+^ ions (Fig. 3F).

Fig. 3N displays the UV-vis spectra of AuNSs prepared *via* the surfactant-free route and coated with different volumes of AgNO_3_. In this case, the SPR peaks were found to be broader and blue-shifted from 745 nm to 610 nm with an increase in Ag^+^. This was accompanied by a decrease in peak intensity, and this difference in optical properties indicates the transformation in spike shape after covering with Ag. The morphology of AuNS-FS after being covered with Ag was also analyzed and compared with AuNS-CTAB@Ag. As shown in Fig. 3J–L, particles were formed with more spikes than AuNSs-CTAB@Ag; however, only small spikes emerged after Ag coating, the core apparently grew bigger, and no polyhedral shapes were detected. Thus, it can be observed that in both the AuNSs-CTAB and AuNSs-FS particles, the adsorption of silver atoms preferentially originated from the spherical core and formed the shell.

**Fig. 4:**
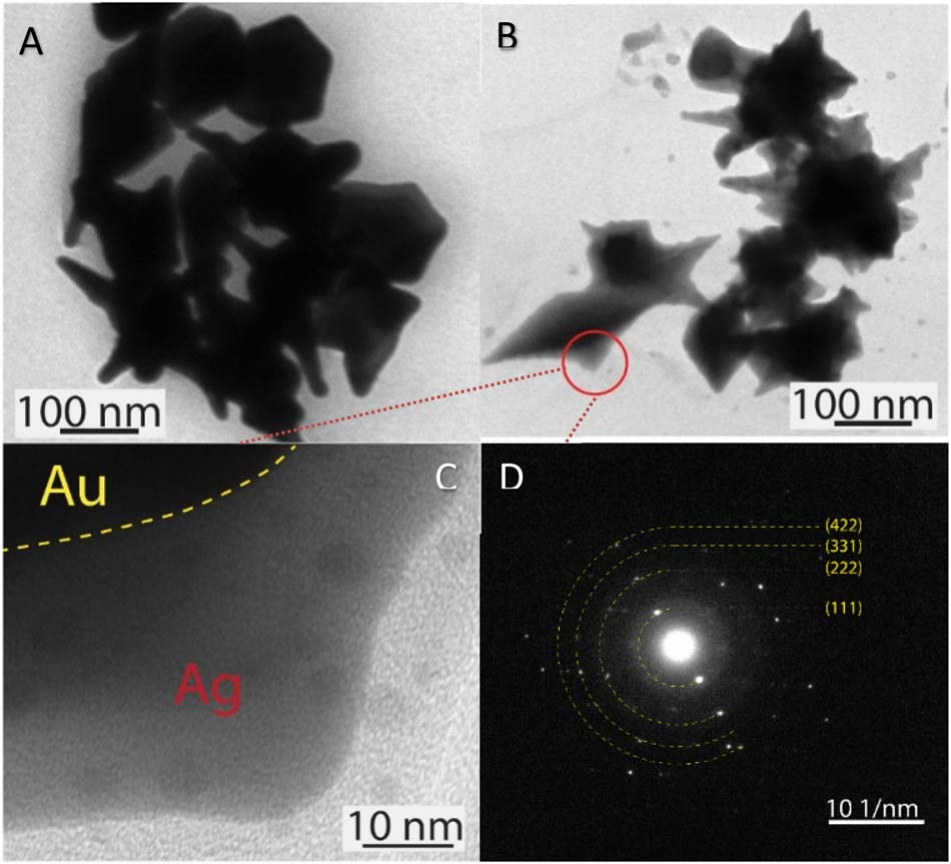
TEM micrographs of AuNSs-CTAB@Ag with an average size of approximately 150 nm covered with (A) 150 and (B) 200 μL of AgNO_3_. (C) HRTEM image exhibiting contrast areas collected on the focus region of one particle reported in (B), revealing the Ag layer coverage on the Au core surface. (D) Selected area electron diffraction (SAED) pattern of AuNS-CTAB@Ag nanocrystal with bright spots, indicating that the formed nanoparticles were highly crystalline.

**Fig. 5:**
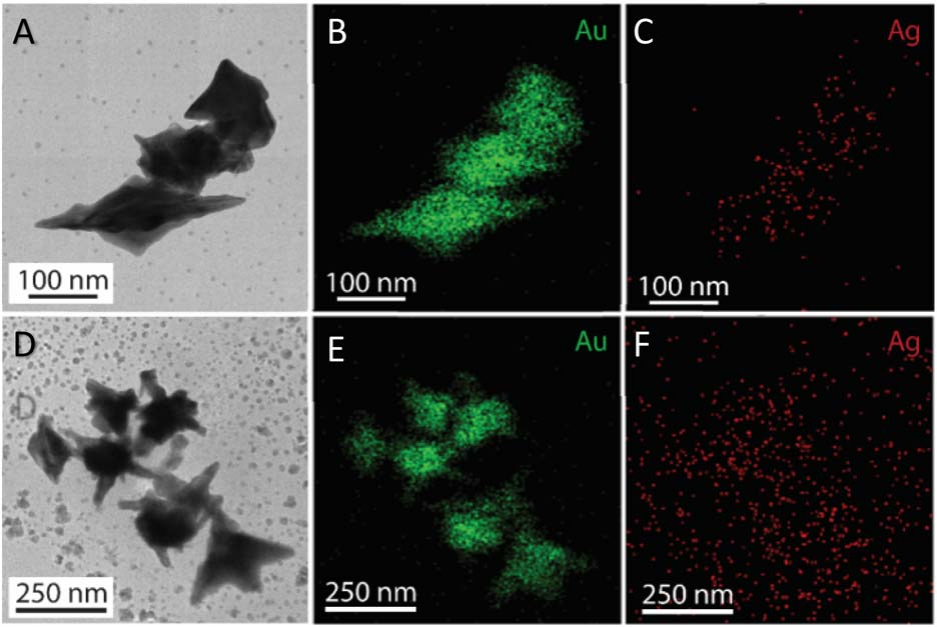
(A) and (D) TEM images of AuNSs-CTAB@Ag at 1.0 and 1.5 mL volume of ethanol added into the two phases dispersion (scale bars of 100 and 250 nm), relatively. Corresponding STEM-EDS mapping for (B) Au and (C) Au elements acquired at a selected area on AuNSs-CTAB@Ag (scale bars, 100 nm), (E) and (F) after adding 1.5 mL ethanol (scale bars 250 nm), respectively.

The morphology transformation of AuNS-CTAB after covering with Ag was further analyzed by TEM. Fig. 4A illustrates the morphologies of AuNS-CTAB@Ag synthesized using 200 μL of 10 mM AgNO_3_. The spikes became more challenging to distinguish with a larger core than the pristine AuNSs, and several particles appeared like nano bipyramids when observed from the lateral sides (Fig. 4B). Furthermore, Fig. 4C exhibits the HR-TEM micrograph of selected areas on the AuNSs-CTAB@Ag, clearly showing the core–shell structure with the AuNS core covered with a silver layer. This is typically based on the high contrast between the two regions, including the dark part assigned to the Au core and the brighter part related to the Ag layer. The selected area electron pattern (SAED) analysis of the AuNS-CTAB@Ag particles assigned the diffraction spots to the fcc crystalline structure of the nanoparticles. The interplanar spacing values evaluated from the diffraction pattern were 2.41, 1.18, 0.94, and 0.83 Å, corresponding to the (111), (222), (331), and (422) planes of fcc,^72^ respectively. These results are consistent with the reference value of JCPDF number 04-0784. Fig. 5 exhibits the STEM-EDS mapping of the gold (green scale) and silver (red scale), showing the core–shell structure of the obtained AuNS-CTAB@Ag nanoparticles. The distribution of Ag element was concentrated on the surface of the AuNSs synthesized with CTAB (Fig. 5C and F) and was less than that of the Au core prepared without surfactant (Fig. 7D). This may be due to the fact that the CTAB molecules were still attached to the surface of AuNSs, preventing the coverage of the Ag atoms on the Au core. Also, it was observed that the relative Au atom percentage was about 67.7% and the Ag atom percentage was 32.3% (the corresponding EDS mapping profile is shown in Fig. S7 of the ESI†).

**Fig. 6:**
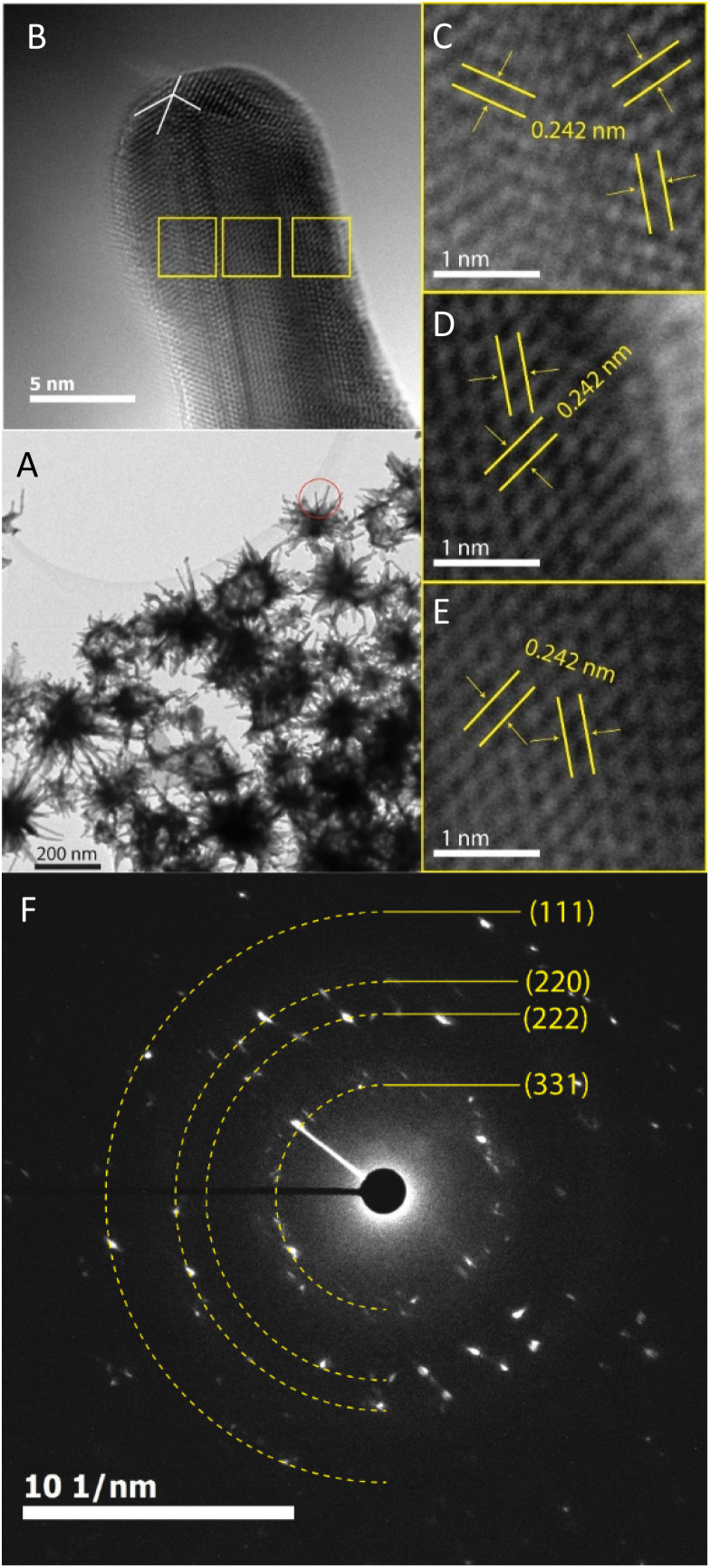
(A) TEM images of AuNSs-FS@Ag with many branches protruding from the core of the two-component nanoparticles and (B) corresponding HR-TEM of the one branch selected from the nanoparticles in (A). (C)–(E) HR-TEM images taken at three selected regions on the branch of AuNSs-FS@Ag, presenting the *d*-spacing of the (111) crystalline facets and (F) SAED pattern of AuNS-FS@Ag nanocrystal with many bright spots, suggesting the crystalline structure of the nanoparticles.

**Fig. 7:**
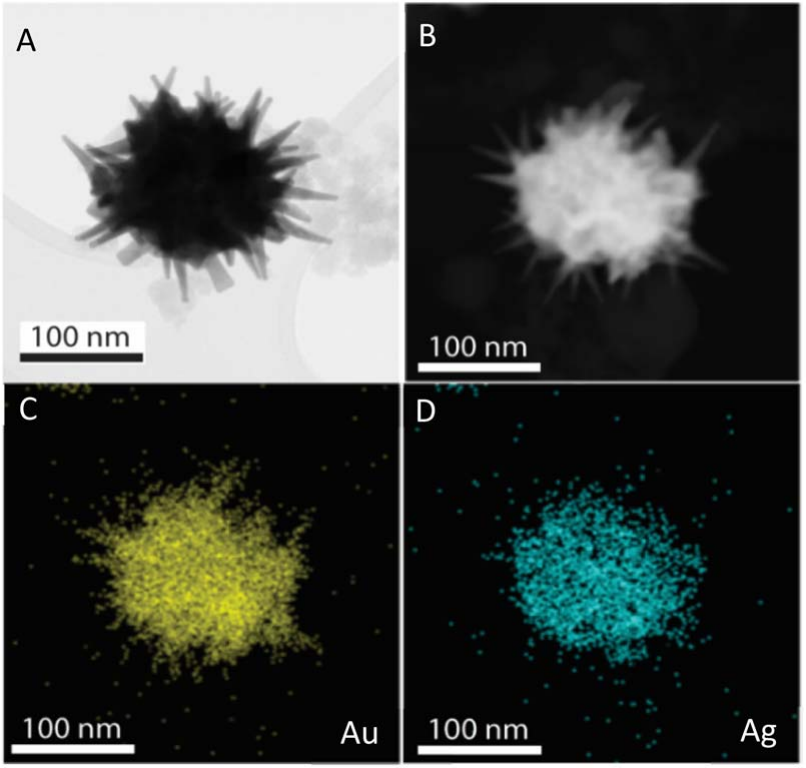
STEM images of AuNSs-FS@Ag taken in (A) bright field and (B) dark field mode. EDS mapping analysis for (C) Au and (D) Ag elements obtained at a selected area on the AuNSs-FS@Ag.

The prepared AuNS-FS@Ag nanoparticles had a core–shell structure. Their core (dark region) was made of AuNSs-FS and was covered with a silver layer (bright region), as shown in Fig. 6A. Additionally, the interplanar spacing of the Au fcc structure was clearly visible in the HR-TEM micrograph of selected areas of the nanoparticle (Fig. 6B–E). The selected area electron pattern (SAED) of the particles displayed a ring shape with many bright spots (Fig. 6F), indicating that they had crystalline structures. An individual AuNS-FS@Ag particle was also studied by STEM and EDS mapping (Au and Ag elemental distribution) to verify the formation of the AuNS-FS@Ag core–shell structure. The characterization results illustrated in Fig. 7C and D show that the AuNS@Ag morphology significantly differed from the structure of the nanoparticles synthesized with CTAB. Specifically, the uneven distribution of Ag on the AuNS surface, primarily concentrated in the urchin-like core and slightly covered on the AuNS tips, revealed that the sharp branches of AuNSs were still exposed to the outside.

**Fig. 8:**
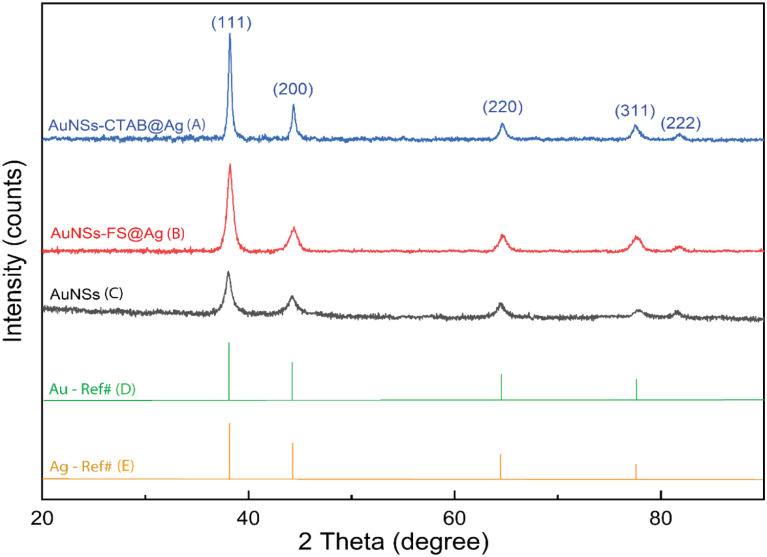
Powder X-ray diffraction patterns of (A) AuNSs, (B) and (C) AuNSs-FS and AuNSs-CTAB after covering with an Ag layer and (D) and (E) Au and Ag reference diffraction peaks from JCPDF data (04-0783 and 04-0784, respectively).

The crystalline structure of AuNSs-FS@Ag and AuNSs-CTAB@Ag were further characterized by XRD (Fig. 8). In their XRD patterns, four high-intensity peaks were observed at the 2*θ* values of 38.18°, 44,42°, 63.00°, and 77.49°, which are assigned to the (111), (200), (220), and (311) planes of the FCC crystalline structure of gold and silver metal, respectively.^72^ Because the Au and Ag metals also possess face-centered cubic (FCC) structures and similar peaks located at Bragg's angles based on the JCPDF card numbers 04-0783 and 04-0784, respectively, it was challenging to distinguish the diffraction peaks of Au and Ag, as shown in Fig. 8, with only one group of diffraction peaks.

### 2.3. Preparation of nanoarray at the interface of oil–water phases

The nanoarray was prepared *via* the self-assembly of the AuNS@Ag particles at the interface of two phases, *i.e.*, oil and water. The two-phase oil–water-forming agents used in this study are ethanol and cyclohexane. Ethanol was dissolved in water and added to the colloidal solution to form a homogeneous phase (water phase) before the addition of cyclohexane to the above solution. Given that cyclohexane is insoluble in water or ethanol, it formed a separate phase (oil phase) on the surface of the ethanol and water phases. Due to the electrostatic interactions between the nanoparticles,^73^ the AuNS@Ag particles self-assembled and formed a large cluster between the oil and water phases.

In the case of AuNSs@Ag prepared using CTAB, they were hardly arranged at the interface of the two phases because the CTAB could not be totally removed from AuNSs-CTAB@Ag. Specifically, emulsification occurred after stirring the colloid with cyclohexane. Thus, AuNS@Ag-CTAB could not be fused into a uniform array, which can be attributed to the double-layer micelle structure of the CTAB molecule,^74,75^ inhibiting the aggregation of AuNS@Ag at the oil–water interface. The orientation of AuNSs@Ag adsorbed on the water/oil interface is mainly influenced by the reduction in interfacial energy and the size of the nanoparticles.^41^ The small-sized particles could not be strongly confined at the water/oil interface, thus influencing the stability of the formation of the membrane-like structure.^41^ Based on the above-mentioned results, we predicted that the AuNS@Ag synthesis pathway plays a vital role in the self-assembly of the nanoparticles at the interface between the two phases. The use of too much CTAB in the synthesis process of AuNSs leads to the emulsification effect. In this case, the attraction of the nanoparticles to each other may decrease, resulting in a reduction in the migration of the nanoparticles at the w/o interface.^46,76^

The analytical results from the SEM images in Fig. 10 show that the AuNS-CTAB@Ag nanoarray obtained by the self-assembling method had a relatively uniform particle distribution and not locally clustered, which were separated by the centrifugation method. The average size of the AuNS particles is about 120 nm, with 5–9 independent branches (Fig. 10A), including an average long branch of about 50 nm and an Au core of about 70 nm. After being covered with a silver layer, the AuNS-CTAB@Ag particles grew more unevenly in the core than in the branches, indicating that the silver coating was concentrated mainly in the core (Fig. 10BC). It can be seen that the size of the particle core significantly increased with an average size of approximately 90 to 100 nm. However, the particles remained star-shaped because the branches were not yet been encapsulated by silver. In addition, the water-phase color changed from dark blue with a yellowish tint to light blue when more AuNS-CTAB@Ag nanoparticles assembled at the interface of the two phases (Fig. 10D and E). Typically, the uniform distribution of particles can create a greater contact area for the absorption of analyte molecules, and also creates a large number of “hot-spots” when the AuNSs-CTAB@Ag branches can contact the neighbor particle branches.^77^ However, the SEM images illustrated in Fig. 10G and H show that the particle arrangement density is relatively low, with significant gaps between each particle. This condition is unsuitable for creating sites for improving the electromagnetic field.

When AuNSs-FS@Ag were used to create nanoarrays, the nanoparticles arranged closely due to their unique shape, as shown in Fig. 10I and N. The particles possessed spikes on their surface, causing them to appear tightly interlocked, but there were still small gaps between them (as shown in Fig. 10I). Additionally, as more silver was added to cover the AuNSs, the particle core became larger (as shown in Fig. 10J), causing the nanoparticles to come closer and fold more tightly, ultimately decreasing the voids. In comparison to using AuNSs-CTAB@Ag, the AuNSs-FS@Ag layer spread more evenly on the surface of the glass, as shown in Fig. 10L. Based on the observed images, the arrangement of nanoparticles was denser compared to AuNSs-CTAB@Ag. This density contributed to multiple amplification of the electromagnetic field regions, which is highly advantageous for conducting SERS tests.

The distribution of elements in the AuNS-FS@Ag and AuNS-CTAB@Ag nanoarray structures was also evaluated through the EDS mapping method (Fig. S8†). According to the EDS analysis result illustrated in Fig. S8A–C,† the AuNS-FS@Ag nanoparticles were more homogeneously distributed than AuNSs-CTAB@Ag in the nanoarray structure (Fig. S8D–F†) with solely Au and Ag and absence of other chemical elements. In the case of AuNSs-CTAB@Ag (Fig. S8D–F†), many regions with space not filled by the Au or Ag elements appeared in the EDS mapping images, suggesting an uneven spread of the nanoparticles on the surface of the tested substrate. Additionally, it can be seen in the mapping images of Au and Ag that the Ag element was partly distributed compared to Au, revealing the incomplete coverage of the Ag layer on the AuNS particles.

**Fig. 9:**
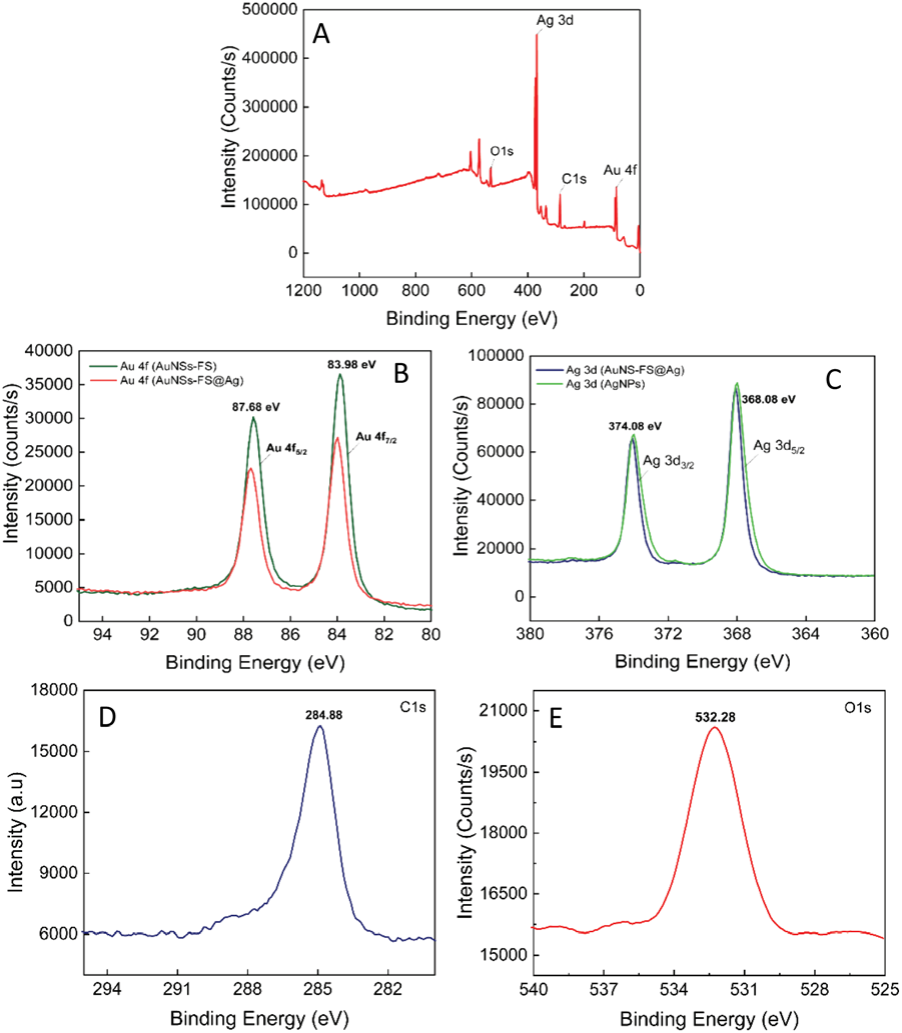
(A) XPS survey spectrum of the AuNS-FS@Ag nanoarrays. High-resolution XPS spectra centered on (B) Au 4f (studied in the AuNS-FS and AuNS-FS@Ag samples), (C) Ag 3d (in the AuNS-FS and AuNS-FS@Ag samples), (D) C1s, and (E) O1s.

The composition of the AuNS-FS@Ag self-assembled nanoarrays was determined by X-ray photoelectron spectral analysis (XPS). The results of the analysis are shown in Fig. 9, with the high-resolution spectra centered on Au 4f displaying two peaks for Au 4f_5/2_ and Au 4f_7/2_ at 87.68 and 83.98 eV, respectively (Fig. 9B). The high-resolution Ag 4d spectra display two peaks at 374.08 and 368.08 eV, indicating the metallic nature of the Ag layer on the star-like core (Fig. 9C). This finding is consistent with previous literature.^78,79^ Additionally, we conducted a parallel comparison of the XPS analysis of the individual AuNS-FS (Fig. 9B, green curve) and AgNP (Fig. 9C, green curve) samples to understand the shift in the peak binding energy. The results indicate a minimal shift in the binding energy compared to AuNSs-FS and AgNPs (please refer to Table S1† for detailed information on the XPS peak positions). The analysis of the XPS spectrum showed a C1s signal at 284.88 eV and an O1s signal at 532.28 eV, which are assigned to C (CH_2_),^80^ suggesting the presence of trisodium citrate, which was employed as the reducing agent in the seed preparation process.

**Fig. 10:**
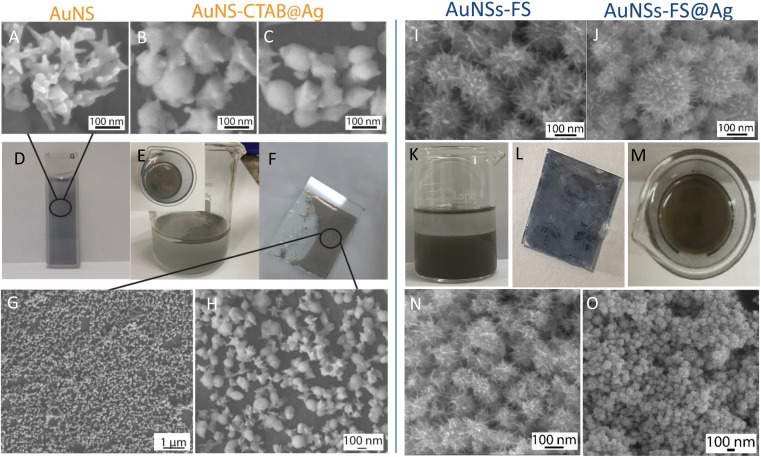
Process for the preparation of AuNS@Ag nanoarray: SEM images of (A) AuNSs prepared with CTAB, (B) and (C) AuNSs-CTAB after being covered with Ag layer, (D) dark blue color of AuNS colloid, (E) AuNS-CTAB@Ag nanoarrays formed at the interface between cyclohexane/water phases, (F) AuNS@Ag nanoarrays transferred to a glass slide, and (G) and (H) SEM images of AuNS@Ag nanoarrays taken at different magnifications (1 and 0.1 μm, respectively). SEM images of the AuNSs synthesized without using CTAB and covered with (I) thin layer of silver, (J) AuNS-FS@Ag formed using a large amount of silver ions, where the particle morphology is different from that prepared with CTAB. (K)–(M) Nanoarray of AuNSs-FS@Ag, from their formation at the oil/water interface to a thin layer on a glass slide. (N) and (O) SEM images of AuNSs-FS@Ag before and after the membrane-like structures are transferred to a glass slide, respectively.

### 2.4. SERS measurement with crystal violet using AuNS-CTAB@Ag nanoarray

**Fig. 11:**
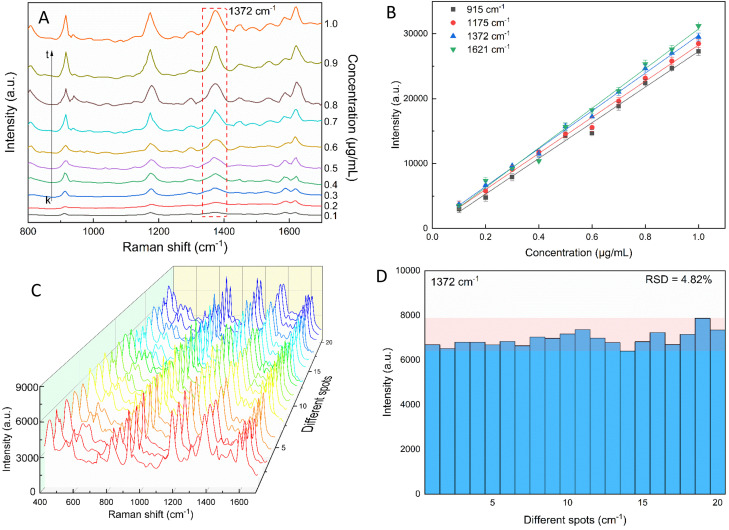
(A) Typical SERS spectra for various crystal violet concentrations tested with AuNS-CTAB@Ag. (B) CV calibration curves of the 915, 1175, 1372, and 1621 cm^−1^ characteristic peaks used to study the relationship between peaks intensity and concentration. (C) SERS spectra of 20 distinct locations on the AuNS-CTAB@Ag nanoarray tested with crystal violet (0.2 μg mL^−1^). (D) SERS intensity of crystal violet at 1372 cm^−1^ from 20 random detection spots in AuNS-CTAB@Ag nanoarray.

The star-like nanostructure could provide a high SERS efficiency upon the generation of local surface plasmons at the core and the tips. Besides, combining the stability of AuNSs with the stronger SPR of the silver shell further improved the SERS effects.^81–83^ The SERS study of CV with the AuNS-CTAB@Ag nanoarray is shown in Fig. 11, where the Raman peaks located at 1621 and 1591 cm^−1^ are assigned to the *C*-phenyl in-plane anti-symmetric stretching.^84,85^ The weak-intensity peak at 727 cm^−1^ is related to the C–N–C symmetric stretching vibration. The peaks that appeared at 801 cm^−1^ and 915 cm^−1^ are attributed to the phenyl-H out-of-plane anti-symmetric bending and phenyl ring breathing mode, respectively.^86^ The *C*-phenyl and C–H in-plane anti-symmetric stretching is represented by the peak located at 1175 cm^−1^.^85^ The peak at 1372 cm^−1^ is assigned to the C–N, phenyl-*C*-phenyl anti-symmetric stretching.^85,87^ Most of the experimental findings are consistent with previous reports, except for minor differences. Fig. 11A illustrates the SERS spectra from serial experiments surveyed on the AuNS-CTAB@Ag nanoarray with different concentrations of CV ranging from 0.1 to 1.0 μg mL^−1^. Apparently, the intensities of CV characteristic peaks at 915, 1175, 1372 and 1621 cm^−1^ concomitantly increased with an increase in the concentration of CV.

In particular, the high-intensity peak at 1175 cm^−1^ was assigned to *C*-phenyl, and the CH in-plane antisymmetric stretching was selected as the prominent characteristic peak. Fig. 11B illustrates the Raman intensities of the peaks at 915, 1175, 1372, and 1621 cm^−1^*versus* low CV concentrations ranging from 0.1 to 1.0 μg mL^−1^. The high linear fit curve (*R*^2^ = 0.994) for the strong Raman characteristic peak at 1372 cm^−1^ studied in the concentration range of 0.1 to 1.0 μg mL^−1^ suggests the good quantification of CV. The limit of detection (LOD) for CV was 0.044 μg mL^−1^ and the limit of quantification (LOQ) was 0.134 μg mL^−1^, which were calculated based on eqn (2) and (3), respectively. The analysis data are shown in detail in Table S2.† In summary, the results further demonstrate that the prepared AuNS-CTAB@Ag substrate can improve the SERS performance.

### 2.5. Reproducibility of the self-assembled AuNS-CTAB@Ag nanoarray

The reproducibility and uniformity of the SERS spectral signal primarily influence the practicability of the AuNS-CTAB@Ag nano-substrate in determining a trace amount of CV. The SERS spectra of CV were recorded at 0.2 μg mL^−1^ using 20 random individual points from 5 substrates to confirm that the nano-substrate can generate high reproducibility at low CV molecular concentrations. Four different points were chosen in an area of 100 μm^2^. As shown in Fig. 11C, the 20 individual Raman spectra of CV with the concentration of 0.2 μg mL^−1^ were similar, with almost no shift in the positions of the characteristic peaks. Furthermore, the relative standard deviation (RSD) was determined to evaluate the reproducibility of the SERS nano-substrates. The results show that the AuNS-CTAB@Ag substrate has good reproducibility and the RSD value from 20 points at 1175 cm^−1^ is 5.76%. Moreover, the intensities of the Raman peaks at 915, 1175, and 1621 cm^−1^ are illustrated in Fig. S9† with the corresponding RSD value of 7.75%, 5.54%, and 7.10%, respectively. These results prove that the as-prepared AuNS-CTAB@Ag nano-array has good reproducibility.

### 2.6. SERS nanoprobes with crystal violet using AuNS-FS@Ag nanoarray

**Fig. 12:**
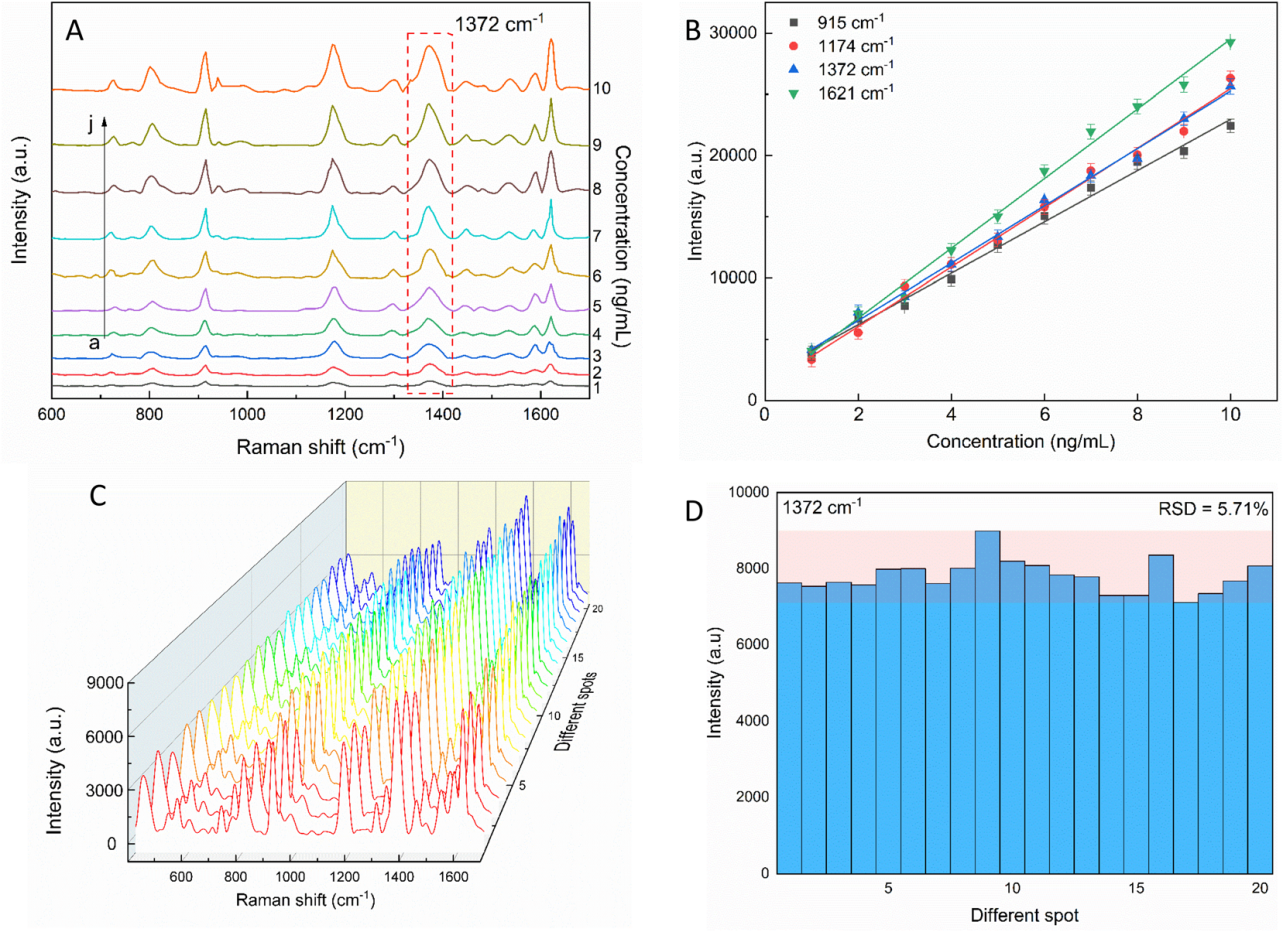
(A) SERS spectra of various crystal violet concentrations tested with AuNS-FS@Ag without CTAB. (B) Corresponding linear calibration plot between CV primary peak intensity and different CV concentrations. (C) SERS spectra of 2 ng mL^−1^ CV at 20 distinct points on AuNS-FS@Ag (4 spectra for each substrate). (D) Intensity of the characteristic Raman peak of 2 ng mL^−1^ CV at 1372 cm^−1^ and the corresponding RSD value.

The sensitivity of the SERS performance was further studied with AuNSs-FS@Ag under the same conditions as AuNS-CTAB@Ag for comparison. The results of the SERS analysis, as shown in Fig. 12A, prove that the trace concentrations of the analyte were well detected on the AuNS-FS@Ag nano-substrate. The ability of AuNSs-FS@Ag to enhance the SERS performance was better than that of AuNSs-CTAB@Ag, with the increase in sharp Raman peaks correlated with an increase in CV concentration. The locations of the characteristic peaks at 1174, 1372, 1621, and 915 cm^−1^ showed negligible deviations from the spectra obtained from AuNSs-CTAB@Ag. Besides, these peaks were additionally plotted with a low concentration ranging from 1 to 10 ng mL^−1^ (Fig. 12B). The Raman signal at 1372 cm^−1^ was recorded due to the high linear fit curve (*R*^2^ = 0.996) of the calibration curve studied at the low concentration (1.0–10 ng mL^−1^). This result suggests the high measurement sensitivity for the trace detection of CV, further proving the better enhancing SERS performance ability of AuNSs-FS@Ag compared to the AuNS-CTAB@Ag nano-substrate. The LOD and LOQ values for CV using the AuNS-FS@Ag nanoarray were 0.45 ng mL^−1^ and 1.38 ng mL^−1^, respectively. It can be seen that these values are about 100-fold smaller than that obtained on the AuNS-CTAB@Ag nanoarray. Based on the results obtained with the AuNS-FS@Ag nanoarrays at low CV concentrations, it can be seen that these nano-substrates obviously exhibited greater sensitivity than the AuNS-CTAB@Ag nanoarrays.

### 2.7. Reproducibility and stability of the AuNS-FS@Ag nanoarrays

The repeatability measurement at 20 random points on the AuNS-FS@Ag substrate at the same CV concentration of 2.0 ng mL^−1^ also showed excellent reproducibility with the RSD of 5.71%. The excellent SERS reproducibility behavior of the AuNS-FS@Ag nanoarray can be attributed to its uniformly arranged structure (Fig. 12D). The Raman intensities of the characteristic peaks at 915, 1174, and 1621 cm^−1^ and corresponding RSD values are further illustrated in Fig. S10.† Additionally, the stability of the self-assembled nanoarrays was evaluated to further develop the practical applicability of the SERS nano-substrate. In detail, the SERS performance of the self-assembled AuNS-FS@Ag nanoarray substrates studied for 2.0 ng mL^−1^ CV was determined at weekly intervals. The AuNS-FS@Ag nanoarrays were kept at room temperature and selected randomly from various substrates made using similar synthesis methods (Fig. S11†). Based on the Raman analysis, it was found that the peaks detected at 915, 1174, and 1372 cm^−1^ maintained around 90% of their original intensity in the nanoarray substrates even after 42 days of storage at room temperature.

### 2.8. Enhancement factor (EF) determination

The EF values were estimated to compare the SERS performance between the AuNS-CTAB@Ag and AuNS-FS@Ag nano-substrates. Typically, the EF factor was calculated based on eqn (1), as follows:^85,88^1
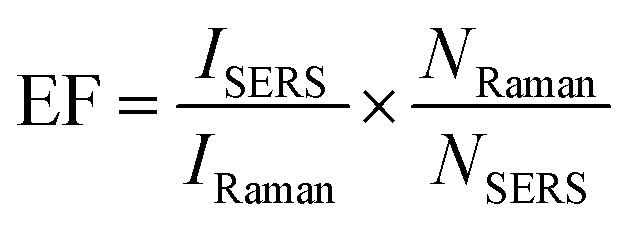
where *I*_SERS_ and *I*_Raman_ denote the intensities of the SERS peaks of 0.02 μg mL^−1^ and 1000 μg mL^−1^ concentration of CV. *N*_Raman_ and *N*_SERS_ are the number of molecules on the silicon wafer Raman substrate and AuNSs-FS@Ag. The intensities were collected at the Raman shift of 1372 cm^−1^ to estimate the EF values. *N*_Raman_ was determined by dropping CV on a silicon wafer, while *N*_SERS_ was measured with AuNSs-FS@Ag (or AuNSs-CTAB@Ag). The EF value for AuNS-FS@Ag calculated based on eqn (1) is 1.2 × 10^7^, while that for AuNSs-CTAB@Ag is 3.43 × 10^5^. These EF values indicate that AuNS-FS@Ag exhibited significantly higher enhancement ability than the AuNS-CTAB@Ag substrate. The procedure for the determination of EF is described in detail in the ESI.†

### 2.9. Detection of CV in real natural water samples

**Fig. 13:**
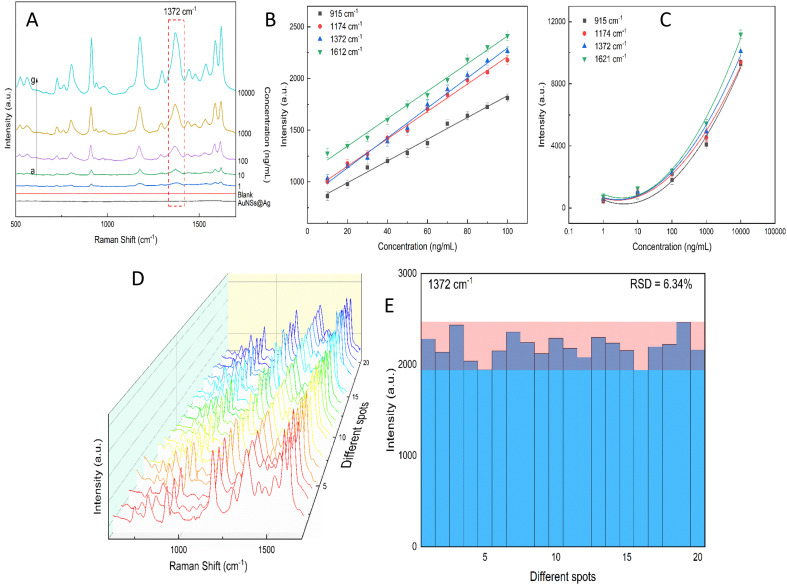
(A) SERS spectra of different CV concentrations ranging from 1 to 10^5^ ng mL^−1^ spiked in Dong Nai River water and further investigated with the AuNS-FS@Ag self-assembled nanoarrays. CV calibration curves of 915, 1174, 1372, and 1612 cm^−1^ characteristic peaks to determine the relationship between peak intensity and concentration ranging from (B) 1.0 to 100 ng mL^−1^ and (C) 1.0 to 10^5^ ng mL. (D) SERS spectra of 20 different spots on the AuNS-FS@Ag nanoarrays examined using CV at a concentration of 100 ng mL^−1^. (E) Intensity of the CV peaks at 1372 cm^−1^ measured at 20 different detection locations on the AuNS-FS@Ag nanoarrays.

Excessive amounts of crystal violet in natural water can pose a significant risk to human health, including acute toxicity if ingested, severe eye damage,^89^ and even cause cancer.^90^ Also, crystal violet is highly hazardous to the aquatic environment and can cause acute and chronic harm.^90^ Thus, a rational and convenient approach to detect CV in real water samples with high sensitivity and reproducibility is essential. The developed method should provide accurate and consistent results with high sensitivity. The characteristic Raman peaks in Fig. 13A show an increase in intensity with an increase in the concentration of CV. The AuNS-FS@Ag substrate has a low LOD of 6.07 ng mL^−1^ for CV in natural water samples and a limit of quantification (LOQ) of 18.4 ng mL^−1^, which was calculated based on the linear relationship between the intensity of the peak at 1372 cm^−1^ and the concentration of CV, which ranged from 10 to 100 ng mL^−1^ (Fig. 13B and C). The developed SERS self-assembled nanoarray has high potential for quantifying CV in natural water samples and other organic compounds, as supported by its *R*^2^ value of 0.963. The reproducibility of the SERS nano-substrates was also tested. As depicted in Fig. 13D and E, the Raman signals remained stable with an RSD of 6.34% after measuring 20 individual spots on five nano-substrates prepared under similar conditions. These findings demonstrate that the substrate is suitable for testing natural samples.

### 2.10. Computation of the role of CTAB in the Au@Ag structure

The SERS enhancement ability of a nanomaterial depends on its composition, structure, and morphology.^91–93^ The presence of CTAB molecules can affect the Raman spectrum of the core–shell AuNS@Ag structure. Wu Zhou *et al.*^94^ suggested that surface plasmon resonance in nanocluster systems can be enhanced locally at the atomic scale and used in the development of atomic-scale nanoplasmonic and quantum plasmonic devices. Furthermore, Oscar A. Douglas-Gallardo *et al.*^95^ concluded that the atomic-scale DFT method can well-describe the plasmonic properties of Au nanoclusters. Due to its high-quality growth, chemical stability, and tailorable optical properties, the core–shell structures of AuNS-coated Ag layers with and without CTAB are reasonable systems to study plasmonic properties at the atomic level of theory.

**Fig. 14:**
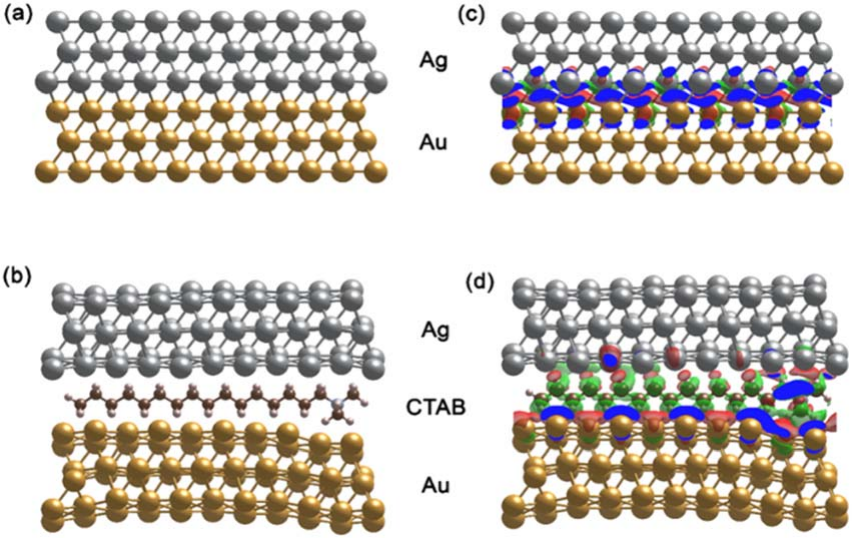
(a) and (b) Optimized Au@Ag structure without and with CTAB molecule, respectively. (c) and (d) Differential charge density at the interfaces for Au@Ag without and with CTAB molecule. The red and green isosurfaces indicate charge accumulation and depletion, respectively. The isosurface level is set at 0.001 e^−^ A^−3^.

According to our experiment, the AuNSs and AuNSs stabilized with CTAB were covered with Ag layers, and hence in the structure of AuNSs@Ag with CTAB, CTAB is in the middle as a sandwich structure with the fcc (111) surfaces of AuNSs and thin Ag layers. Assuming that these molecules are adsorbed on the gold surface through the bromide ions, in principle it is possible to identify up to three different conformations, where two of them will by oriented parallel to the gold surface, whereas the other one featured a perpendicular cavity.^96^ According to the study by Faeli Qadikolae *et al.*,^97^ the alkyl tails of CTAB molecules prefer to align parallel to each other to maximize their interactions. Also, the CTAB surfactant molecules with an extended backbone explore a wide range of conformations upon adsorption including preferred orientations in direct contact with the Au (111) surface.^98^ Based on the aforementioned points from previous studies, we ensured that our systems at the atomic level of AuNSs@Ag without and with CTAB located in the middle had well-defined plasmonic properties, especially the SERS of the AuNS@Ag and AuNS-CTAB@Ag nanoparticles. Therefore, to investigate the effect of the CTAB molecule on the structural morphology and plasmonic properties of AuNSs@Ag, initially we compared the optimized structure of the AuNS@Ag films without and with the CTAB molecule, as depicted in Fig. 14(a) and (b), respectively.

The AuNS@Ag film without the CTAB molecule, as shown in Fig. 14(a), exhibits a more uniform distribution and alignment of the Au and Ag atoms, where the fcc (111) structure it retained. Meanwhile, AuNSs@Ag with the CTAB molecule shows strongly bent Au and Ag layers, as shown in Fig. 14(b). Several studies reported that the film roughness strongly influences the plasmonic properties of metal nanostructures, significantly affecting the Raman scattering intensity.^99,100^

Moreover, CTAB plays a role as a stabilizer in the colloidal dispersion by protecting the gold from aggregation or dissolution. The aggregation behaviour and linear self-assembly mechanism of citrate-stabilized gold colloids was provoked by the addition of CTAB.^101^ In this case, a very high concentration of CTAB serves as a stabilizer to prevent the agglomeration of the gold NPs, while a low concentration of CTAB serves as a cross-linker for the linear self-assembly of AuNSs. Besides, the nanoparticles used for self-assembly carry a surface charge, which is important because the resulting interparticle electrostatic repulsion prevents their aggregation.^102^

In addition, CTAB is a cationic surfactant, which can form a self-assembled monolayer at the interface between the Ag and Ag layers. This leads to a change in the electronic structure of AuNSs@Ag. Fig. 14(c) and (d) demonstrate the differential charge density for the AuNSs@Ag film without and with CTAB, where the red and green isosurfaces indicate charge accumulation and depletion, respectively. Overall, there is charge redistribution at the interfaces. In Au@Ag without CTAB, the charge accumulation is located between the Au and Ag interfacial layers. However, in AuNSs@Ag with CTAB, the charge accumulation only intensively takes place in the Au interface layers. Table 1 provides the DFT-calculated Bader charge analysis of AuNPs, Ag layers, and CTAB of AuNSs@Ag and AuNSs-CTAB@Ag. The sum charge transfer of AuNPs represented by the Au (111) surfaces was computed to be 1.66*e* in AuNSs@Ag, whereas it decreases to 0.73*e* in AuNSs-CTAB@Ag. The presence of CTAB accounts for −0.41*e* charge transfer along with −0.32*e* from Ag layers of the total charge, affecting the Au surface charge in AuNSs-CTAB@Ag. Hence, our calculations prove that CTAB influences the self-assembly behaviour and SERS of the AuNP@Ag nanoparticles, which is consistent with the experimental results.

**Table 1:** The DFT-calculated Bader charge analysis of AuNPs, Ag layers and CTAB of AuNSs@Ag and AuNSs-CTAB@Ag

	AuNSs@Ag		AuNSs-CTAB@Ag		
	AuNPs	Ag layers	AuNPs	CTAB	Ag layers
Charge transfer (*e*)	1.66	−1.66	0.73	−0.41	−0.32

## 3. Conclusions

In this work, AuNS-FS@Ag and AuNS-CTAB@Ag nanoarrays were successfully prepared using the oil/water interface self-assembly method for developing SERS nano-substrates. The AuNSs synthesized without using the surfactant possessed numerous tips compared to the AuNSs prepared with CTAB. This morphology is beneficial for developing SERS substrates given that it can generate numerous hot spots on the surface and a gap between adjacent nanoparticles. Our research showed that creating a nanoarray structure from AuNSs-FS@Ag was easier than with AuNSs-CTAB@Ag using the oil/water interface assembly method. Additionally, the AuNS-FS@Ag nanoarray showed a better SERS performance with a low LOD for CV at 0.45 ng mL^−1^ and LOQ at 1.38 ng mL^−1^. The prepared AuNSs-FS@Ag had a good EF value of 1.2 × 10^7^ and remained stable even after 42 days of storage under ambient conditions. This substrate could detect CV in natural water samples from the Dong Nai River with an LOD as low as 6.06 ng mL^−1^ and LOQ of 18.4 ng mL^−1^. Thus, it is expected to be useful for detecting other dyes commonly found in wastewater from the textile industry. Based on the computational results, it is evident that the presence of CTAB on the interfaces of Au@Ag has a significant effect on the local field surrounding the metal nanostructure interface.

## 4. Experimental

### 4.1. Reagent

Tetrachloroauric(iii) acid trihydrate (HAuCl_4_·3H_2_O, 99.5%), trisodium citrate dihydrate (TSC, 99.0%), silver nitrate (AgNO_3_, 99.8%), sodium borohydride (NaBH_4_, 96.0%), l(+)-ascorbic acid (AA, 99.0%), *n*-hexane (C_6_H_14_, anhydrous, >99%), and DI water (>18 MW, Millipore, conductivity <4.3 μS cm^−1^) were purchased from Sigma-Aldrich (Darmstadt, Germany). Cetyltrimethylammonium bromide (CTAB, 99.0%) was purchased from HIMEDIA (Mumbai, India). Crystal violet (C_25_N_3_H_30_Cl, 99.0%) was purchased from AK Scientific, Inc. (Union City, USA). The above-mentioned chemicals were analytical grade and used without further purification. All experiment solutions were prepared using deionized water (DI). Before synthesizing the nanoparticles, glassware and stirring bars were washed with aqua regia water (HCl : HNO_3_, optimal in a molar ratio of 3 : 1), and then thoroughly washed with deionized water.

### 4.2. Synthesis of gold nanostars (AuNSs) using CTAB surfactant

#### Preparation of seed nanoparticles

The Au seed nanoparticles were synthesized *via* the citrate reduction method by reducing HAuCl_4_ with NaBH_4_ at ambient temperature. Firstly, 25 μL of 0.1 M TSC was added to 9.90 mL of 0.25 mM HAuCl_4_ aqueous solution, followed by stirring at 600 rpm for 30 s. Then, 30 μL of 0.1 M NaBH_4_ (at the temperature of 4.0 °C) was rapidly added to the above mixture. Subsequently, the color of the solution changed from transparent yellow to purple-red, indicating the formation of Au nanoparticles in the colloid. After stirring for 2 min, the reaction mixture was left undisturbed for 2 h to disintegrate the excessive NaBH_4_ and was best employed in the next 6 h.

#### Growth process of seeds to form AuNSs

Initially, the growth solution containing 0.25 mM HAuCl_4_, 0.10 M CTAB, and 0.05 mM AgNO_3_ was prepared at room temperature and best used within 24 h. The gold nanostars were formed according to the typical procedure, as follows: 0.8 mL of seed solution was added to 10 mL of as-prepared growth solution and stirred for 20 min at 800 rpm. Then, 50.0 μL of 0.1 M ascorbic acid was rapidly added to the mixture, and the color of the solution changed from transparent yellow to colorless or very pale pink. The mixture was continuously stirred for 30 s. The color of the solution slowly changed from colorless to blue-violet, and gradually became darke blue, indicating the formation of star-shaped gold nanoparticles. The colloidal solution samples were centrifuged at 6000 rpm for 10 min to separate the AuNS particles from the colloidal solution, and the supernatant was substituted with DI water. This washing protocol was repeated three times to remove the unreactive precursors and excess surfactants. Then the obtained sediment containing AuNS particles was redispersed in 5 mL of DI water (solution A).

### 4.3. Synthesis of surfactant-free AuNSs (AuNSs-FS)

The method for the surfactant-free synthesis of gold nanostars was previously proposed by Yuan *et al.*^19^ However, we modified this preparation route to produce AuNSs with an increased number of spikes compared to their particles.

#### Seed colloidal solution

1.5 mL of 1.0% (w/v) trisodium citrate was added into 10 mL volume of 1.0 mM HAuCl_4_, boiled at 60 °C, and gently stirred for 2 min. Then, the obtained solution was filtered through a nitrocellulose membrane with a pore size of about 0.22 μm.

#### The growth of AuNSs in the absence of surfactant

10 mL of 0.25 mM HAuCl_4_, kept at 4 °C, was added to a 100 mL beaker. Afterward, 100 μL of the seed colloid, stored at 4 °C, 10 μL of 1.0 M HCl, and 30 μL of 0.01 M AgNO_3_ aqueous solution, were dropped slowly in the above reaction solution, respectively. The reaction mixture was cooled at 18 °C and magnetically stirred at 200 rpm. Subsequently, the gold nanostars were formed by rapidly adding 40 μL of 0.1 M ascorbic acid to the above mixture. The addition of ascorbic acid to the reaction system had to be fast; otherwise, particles with a spherical or pseudo-spherical morphology would be formed. The color of the colloid changed from transparent to dark blue, suggesting the formation of AuNSs.

### 4.4. Covering of gold nanostars with the silver layer (AuNS@Ag)

Briefly, 275 μL of 10 mM ascorbic acid was added to 5.0 mL of solution A and stirred at 500 rpm for 15 min. Then, 200 μL of 10 mM AgNO_3_ was slowly added to the above mixture at a rate of 20 μL per 30 s, while continuously stirring the mixture for 20 min for the AuNS particles to be covered by an Ag layer, resulting in a colloidal AuNS@Ag solution (solution B). The as-prepared colloid was centrifuged at 6000 rpm and rinsed twice with DI water to remove the unreacted reagent and improve the long-term stability of AuNS@Ag.

### 4.5. Preparation of self-assembled AuNS@Ag nanoarrays

**Fig. 15:**
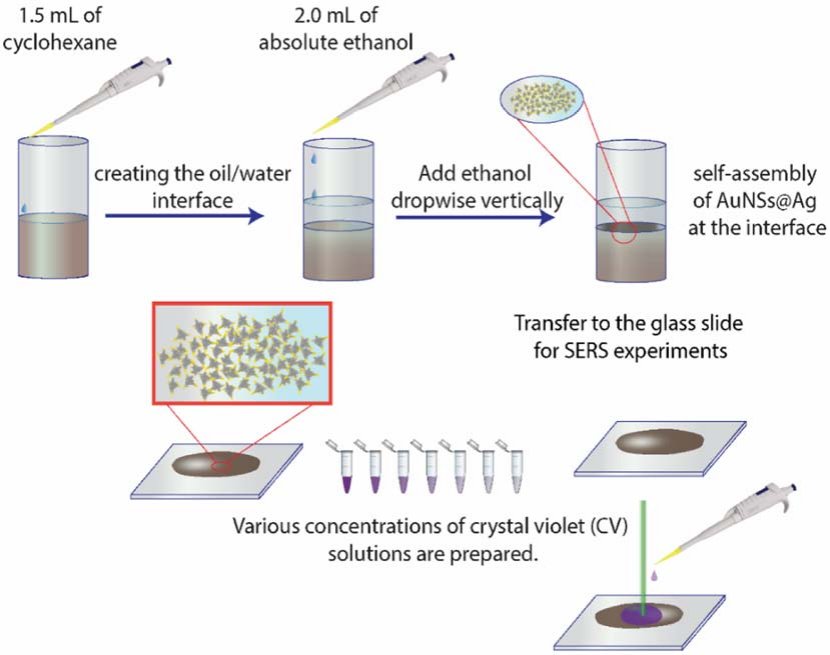
Process for the preparation of the AuNS@Ag nanoarray at the cyclohexane/water interface using the LLISA method for SERS experiments.

Typically, 1.5 mL of cyclohexane was slowly added to 5 mL of solution B to create two separate phases. Subsequently, 2.0 mL of ethanol was vertically injected into the above mixture to pass through the interface between the two phases. The AuNS@Ag particles self-assembled and created a monolayer between the interface of the aqueous and organic phases. This layer could be simply transferred from the interface between the water and organic phases to a glass slide. Then, the cyclohexane and water were allowed to evaporate naturally at ambient temperature (Fig. 15).

### 4.6. Sample preparation for SERS characterization

Crystal violet was employed as the probe molecule to determine the SERS enhancement effects and reproducibility of the AuNS@Ag nano-substrate. A stock solution of crystal violet (100 μg mL^−1^) was prepared in ethanol before diluting to serial concentrations ranging from 0.1, 0.2, and 0.3 to 1.0 μg mL^−1^ for investigation with AuNS-CTAB@Ag, and from 1 to 10 ng mL^−1^ for AuNS-FS@Ag. The pH of the separate solution was maintained at 4.0 using HCl 0.1 M. Besides, microscope glass slides were dipped in aqua regia (HNO_3_/HCl, volume ratio 3 : 1) for 2 h and washed again with DI water before being used. To create the test samples for SERS characterization, we placed the AuNS-CTAB@Ag or AuNS-FS@Ag self-assembled nanoarrays on a clean glass slide. Next, we air-dried the arrays to make a thin film substrate and remove any remaining ethanol through vaporization. Later, 50 μL of crystal violet solution with different concentrations was dropped onto this thin film and naturally dried at ambient temperature for 10 min.

### 4.7. Characterization

UV-Vis spectra were obtained using a UV-Vis-NIR-V670 spectrophotometer (JASCO, Japan). The samples were measured at a scanning rate of 200 nm per minute between 300 and 800 nm, using 1 cm path-length quartz cuvettes. The structural morphology, dispersion, and correlation between the particle shape and optical properties of AuNS and AuNS@Ag were studied *via* scanning electron microscopy on a JEOL JSM-7600F microscope (USA). The size of the nanoparticles in the colloidal suspensions was determined by dynamic light scattering (DLS) performed on a Horiba SZ-100 (Horiba Ltd, Japan). In the DLS characterization, the colloid samples were prepared at a low concentration to avoid multiple scattering effects. The crystalline structure information of AuNSs and AuNS@Ag was provided by the XRD technique using a D8 Advance-Bruker, Germany, with Cu-Kα radiation in the 2*θ* range of 30° to 80° (40 kV, 40 mA). Powder samples were employed for the XRD measurement, which were obtained after air-drying the corresponding colloidal solutions. The formed nanoparticle composition was characterized matching the position of its peaks with the reference data from Joint Committee on Powder Diffraction Standards (JCPDF). The transmission electron microscopy (TEM) technique was used to study the nanostructure of AuNSs and AuNSs@Ag with a JEM-1400 microscope (JEOL, Japan), operated at 200 kV. A small amount of AuNS colloid was dropped on copper grids (300-mesh, Ted Pella, Inc, Redding, CA, USA) and air-dried at room temperature. High-resolution transmission microscopy (HR-TEM) analysis was performed to characterize the crystal structure of single particles (mono-crystalline, poly-crystalline, and amorphous nanoparticles) using an FEI Tecnai G2 F20, USA, at an accelerating voltage of 200 kV. The lattice parameter and crystalline structure were analyzed by selected area electron diffraction (SAED), conducted on a JEOL-2100 (JEOL Ltd Japan). X-ray photoelectron spectroscopy was employed for examining the elemental composition and electronic structure of the surface of the AuNS-FS and AuNS-FS@Ag self-assembled nanoarrays. A Thermo Fisher Scientific X-ray photoelectron spectrometer equipped with a mono-chromatic Al Kα X-ray source, operated at the photon energy of 1486.7 eV, was utilized for the chemical composition analysis.

Raman measurement of the samples was performed on a Raman XploRa Plus (Horiba, France), operated at the induced light wavelength of 532 nm and laser power of 50 nm. The spectra of the samples were collected at a magnification of 50× in the wavelength range of 400 to 3500 cm^−1^. The laser power was set at 100% with the exposure time set to 15 s and two-time accumulation. Capillary tubes with an inner diameter of 1 mm were used for sample coupling. Subsequently, the Raman spectra were analyzed using the LabSpec6 software, followed by the noise cancellation process in some cases.

### 4.8. Data analysis

The limit of detection (LOD) was determined from the standard deviation of the intercepts (*S*_*y*_) and the slope of the calibration curve (denoted as *b*) and calculated according to eqn (2), as follows:^103^2
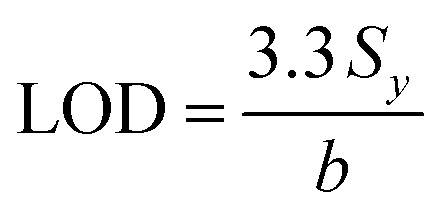


The limit of quantification calculation is also based on the standard deviation of the intercept of the regression line (*S*_*y*_) and the slope value (*b*), as described by eqn (3):^103^3
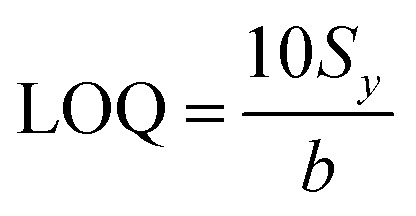


The standard deviation and slope value were obtained from the descriptive statistic data when creating the regression line in the Origin software.

### 4.9. Determination of CV in river water samples

The natural water sample was taken at 10 am from a location near the east bank of the Dong Nai River (Dong Nai Province, Vietnam). The river water sample was first filtered through a UNI-Sci qualitative filter paper with a diameter of 12.5 cm and pore size of 20–25 μm, which was repeated twice before conducting further detection experiments. For spiking the natural water samples, a CV aqueous solution was prepared at a concentration of 100 μg mL^−1^. The testing samples had CV concentrations ranging from 1.0 ng mL^−1^ to 1000 ng mL^−1^. For the SERS experiments, 50 μL of prepared solution was applied to the substrates with the self-assembled AuNS-FS@Ag nanoarray attached. The SERS substrate was left to air-dry at room temperature prior to measurement.

### 4.10. Computational methods

We performed density functional theory (DFT) calculations using the Vienna *Ab initio* Simulation Package (VASP).^104,105^ The interaction between the core and valence electrons was treated by employing the projector-augmented wave (PAW)^106^ method. The generalized gradient approximation (GGA) parameterized by the Perdew–Burke–Ernzerhof (PBE)^107^ was used to describe the exchange–correlation potential. The DFT-D3 correction of the Grimme scheme^108^ was applied for the long-range van der Waal interactions. An energy cutoff of 400 eV was chosen for the wave function expansion. The Brillouin zone was only sampled at the gamma point. Energy convergence was set at 10^−6^ eV, and forces were converged to within 10^−2^ eV Å^−1^.

To investigate the effect of CTAB on the structural morphology and electronic structure of the Au@Ag nanostar, we simulated it using a (2 × 5) rectangular supercell of films consisting of three layers for each atomic type. The films were constructed along the [111] direction because the gold nanostars (AuNSs) were found to preferentially grow along the Au(111) direction.^109^ All layers were fully relaxed. Fig. 14(a) and (b) schematically show the Au@Ag film without and with the CTAB molecule. For all unit cells, the optimized lattice constant of Au of 4.16 Å was used. To avoid artificial interactions between neighbouring slabs, a vacuum distance of 15 Å was applied to all structures.

## Abbreviations

AuNSs: Gold nanostars
AuNSs-FS: Gold nanostars-surfactant-free
AuNSs-CTAB: Gold nanostars-cetyltrimethylammonium bromide
AuNSs-FS@Ag: Gold nanostars-surfactant-free covered by silver layer
AuNSs-CTAB@Ag: Gold nanostars-cetyltrimethylammonium bromide covered with silver layer
TEM: Transmission electron microscopy
CV: Crystal violet

## Author contributions

Conceptualization: Sy Van Vu, Khuong Quoc Vo, Anh-Thu Nguyen, Viet-Ha Le Thi, Nguyet Nhu Thi Pham, In Park; methodology and analysis: Sy Van Vu, Khuong Quoc Vo, Anh-Thu Nguyen; investigation: Anh-Thu Nguyen, Sy Van Vu, Khuong Quoc Vo, In Park, Tien Nu Hoang Lo, Thi Huynh Ho, Nguyet Nhu Thi Pham; writing-original draft: Nguyet Nhu Thi Pham, Khuong Quoc Vo, Sy Van Vu; Writing and editing: Sy Van Vu, In Park, and Khuong Quoc Vo; supervision and editing: Khuong Quoc Vo.

## Conflicts of interest

There are no conflicts to declare.

## Supplementary Material

NA-005-D3NA00483J-s001
