# Supplementary material for: Differences between surfactant-free Au@Ag and CTAB-stabilized Au@Ag star-like nanoparticles in the preparation of nanoarrays to improve their surface-enhanced Raman scattering (SERS) performance
Source: Nanoscale Adv. 2023 Sep 4;5(20):5543–61. doi: 10.1039/d3na00483j (PMC10563836; doi:10.1039/d3na00483j)
Supplement: NA-005-D3NA00483J-s001 [file NA-005-D3NA00483J-s001.pdf]

Electronic Supplementary Material (ESI) for Nanoscale Advances.
This journal is © The Royal Society of Chemistry 2023

Nanoscale Advances

SUPPLEMENTARY INFORMATION

DIFFERENCES BETWEEN FREE-SURFACTANT Au@Ag AND CTAB-STABILIZED Au@Ag STAR-LIKE NANOPARTICLES IN PREPARING NANOARRAYS TO IMPROVE SURFACE-ENHANCED RAMAN SCATTERING (SERS) PERFORMANCE

[a,b]Sy Van Vu, [a,b]Anh-Thu Nguyen, [a,b]Anh-Thi Cao Tran, [a,b]Viet-Ha Thi Le, [c,d]Tien Nu Hoang Lo, [e,f]Thi H. Ho , [a,b]Nguyet. N. T. Pham, [c,d]In Park [a,b]*Khuong Quoc Vo.
*Corresponding authors: In Park, Khuong Quoc Vo

[a.] *Faculty of Chemistry, University of Science, Vietnam National University - Ho Chi Minh City, 227 Nguyen Van Cu Street, Ward 4, District 5, Ho Chi Minh City 70000, Vietnam.*
[b.] *Vietnam National University, Ho Chi Minh City, Vietnam.*
[c.] *Research Institute of Clean Manufacturing System, Korea Institute of Industrial Technology (KITECH), 89 Yangdaegiro-gil, Ipjang-myeon, Cheonan 31056, South Korea.*
[d.] *KITECH school, University of Science and Technology (UST), 176 Gajeong-dong, Yuseong-gu, Daejeon 34113, South Korea.*
[e.] *Laboratory for Computational Physics, Institute for Computational Science and Artificial Intelligence, Van Lang University, Ho Chi Minh City, Vietnam.*
[f.] *Faculty of Mechanical - Electrical and Computer Engineering, School of Technology, Van Lang University, Ho Chi Minh City, Vietnam.*

TABLE OF CONTENTS

Fig. S1. (A) UV-Vis spectrum of the as-prepared AuNSs colloidal samples, (B) and (C) the morphology of formed AuNSs synthesized with CTAB 4

Fig. S2. UV-Vis spectrum of different seed colloid samples investigated the effect of boiling temperature (from 40 to 100°C) in the surfactant-free seed synthesis process approach. TEM images of the obtained Au seed particles prepared at the boiling temperature of (B) 60 ^{o}C, and (C) 90 ^{o}C. 4

Fig. S3. (A) UV-Vis spectrum depicting the effect of different ascorbic acid volumes ranging from 30 to 80 µL (a-f curves) on the formation of star-liked gold nanoparticles. SEM images of the AuNSs-FS synthesized with (B) 30, (C) 40, and (D) 50 µL of ascorbic acid (0.1 M). 6

Fig S4. (A) UV-Vis spectra of the colloidal samples synthesized with difference volumes of precursor $HAuCl_4$ 0.25 mM ranging from 7.0 to 15 mL $HAuCl_4$ (0.25 mM), (B) and (C) SEM images of the obtained AuNSs-FS prepared with 10 and 15 mL of $HAuCl_4$ 7

Please do not adjust margins

Fig S5. FTIR spectrum of the (A) AuNSs-CTAB, (B) pristine CTAB, and (C) the AuNSs after removing unadsorbed CTAB from the solution and a part of CTAB on the surface of the nanoparticle..8

Fig. S7 (A) Overlay EDS signal mapping of Au and Ag elements of the AuNSs@Ag sample synthesized at 200 µL of 10 mM $AgNO_3$, (B) corresponding EDS map summary spectrum shows the atom percentage of Au and Ag...9

Fig. S6 (A) TEM images of AuNSs synthesized at 0.8 mL of seed solution and 10 mL of growth solution, (B) a typical AuNS particle prepared with CTAB at the same condition after removing unadsorbed CTAB through the rinsing process, there is no significant change in the AuNSs shape before and after rinsing, and (C) A relative high-resolution TEM micrograph taken on a selected area of the AuNSs spike, showing the interplanar spacing of 0.24 nm associated with (111) planes in facet-centered cubic. 9

Fig. S8 EDS elemental mapping analysis for the (A) Overlay AuNSs-FS@Ag, (B) Au, (C) Ag distribution of the self-asembled nanoarrays. And, (D-F) the elemental mapping of overlay AuNSs-CTAB@Ag, Au, and Ag distribution in the nanoarrays, respectively. 10

Fig. S9 The SERS intensities of CV at (A) 915 cm^{-1} , (B) 1175 cm^{-1}, and (C) 1621 cm^{-1} from 20 random detection spots from AuNSs@Ag-CTAB nano-substrates and corresponding RSD values...11

Fig. S10 The SERS intensities of CV at (A) 915 cm^{-1} , (B) 1174 cm^{-1}, and (C) 1621 cm^{-1} measured from 20 random detection spots from AuNSs@Ag-FS nanoarray with related RSD values. 12

Fig. S11 (A) SERS experiments on the stability of self-assembled AuNSs-FS@Ag nanoarrays stored after 7, 14, 21, 28, 35, and 42 days and studied on the 2.0 ng/mL of CV aqueous solution. (B) The SERS intensities of the peak at 1372 cm^{-1} were investigated on various AuNSs-FS@Ag nanoarray substrates after 42 days of storage. 13

Table S1. XPS spectrum data with the binding energy (eV) peaks positions for the AuNSs-FS, AuNFs-FS@Ag, AgNPs samples. 13

Table S2. Linear correlation between different concentrations of CV and the intensity of SERS at specific peaks. 13

 This journal is © The Royal Society of Chemistry 20xx

Please do not adjust margins

Please do not adjust margins

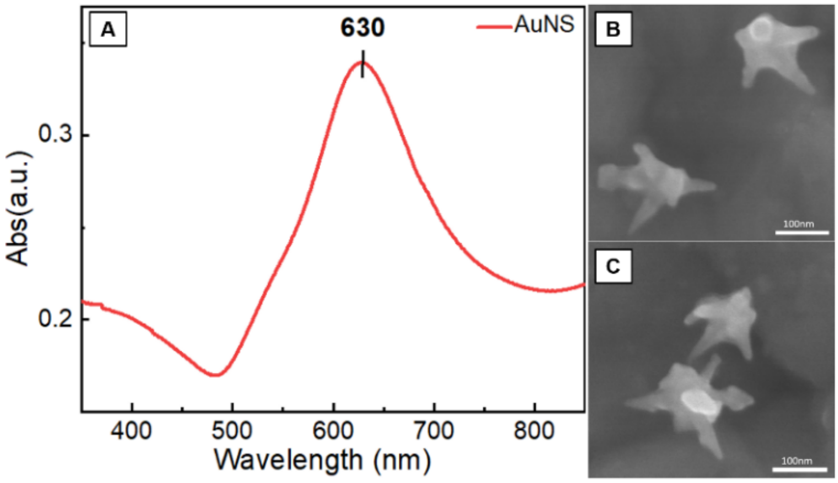

Fig. S1. (A) UV-Vis spectrum of the as-prepared AuNSs colloidal samples, (B) and (C) the morphology of formed AuNSs synthesized with CTAB

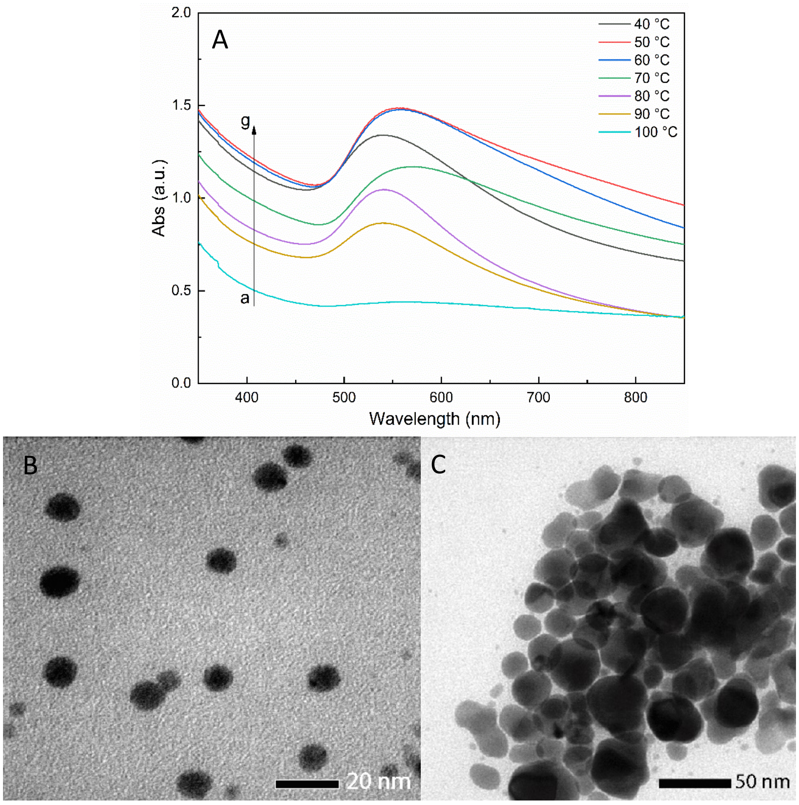

Fig. S2. UV-Vis spectrum of different seed colloid samples investigated the effect of boiling temperature (from 40 to 100°C) in the surfactant-free seed synthesis process approach. TEM images of the obtained Au seed particles prepared at the boiling temperature of (B) 60 ^{o}C, and (C) 90 ^{o}C.

Please do not adjust margins

Please do not adjust margins

Role of Ag ions in the synthesis process of AuNSs-CTAB

AuNSs-CTABs were produced through a seed-mediated technique. During the growth process, the Ag^+ ions are preferentially bound to certain crystalline facets of the seed. This process encourages the attachment of CTAB molecules to the seed crystal surfaces. That reduces the availability of Au ions on these surfaces. In detail, Ag^+ ions are primarily deposited on the (100) crystalline facet, while Br^- ions bind with Ag^+ to create a complex known as [CTABr-AgBr] and get deposited on these gold-selective facets. Thus, this complex impedes the adsorption of Au^{3+} ions. Furthermore, once the [CTABr-AgBr] complex undergoes decomposition, the (100) surface will be predominantly covered by Br^- ions. Subsequently, the gold ion will adsorb more onto the (111) surface, resulting in anisotropic growth of the crystal [1]."

AuNSs-FS synthesis condition for preparing AuNSs-FS@Ag nanoarray

The synthesis condition of AuNSs prepared without using the surfactant and covered with the silver layer was 10 µL of $AgNO_3$ solution 0.01 M, 10 mL of $HAuCl_4$ 0.25 mM, 100 µL of the seed solution, stored at the temperature of 4 ^oC, 10 µL of 1.0 M HCl and 40 µL of 0.1 M ascorbic acid.

For creating two separate layers, 1.5 mL of cyclohexane was added into 5.0 mL of AuNSs-FS@Ag colloid. Then, slowly add 2.0 mL of ethanol to form the interface between the two layers. The particles of AuNSs@Ag will naturally align and create a layer between the organic and aqueous layers. Apply the AuNSs-FS@Ag layer to the glass slides, clean before with aqua regia, and let it dry naturally at room temperature.

Effect of ascorbic acid

In order to gain a deeper understanding of how ascorbic acid affects the formation of star-shaped gold nanoparticles, we kept the $HAuCl_4$ concentration at a fixed concentration of 0.25 mM, used 100 µL seed solution, along with 10 µL of 0.01 M $AgNO_3$ solution. The volume of AA (0.1 M) was varied from 30 to 80 µL. The UV-Vis results observed in Fig S3 indicated that when the addition of AA volume in the growth solution above 40 µL, the SPR of the colloidal solution appeared as a broad band ranging from 650 to 800 nm, it can be inferred that the formation of different nanoparticles shapes in the reaction colloids. However, the ascorbic acid volume of 30 µL yielded an absorption maximum peak at 542 nm (Fig S3) and a further decrease in the absorbance intensity to 0.55, indicating the formation of sphere-like nanoparticles in the reaction system. When the volume of ascorbic acid was reduced from 40 to 30 µL, the star-like morphology decreased, causing the blue shift of SPR peaks from 600 nm to 542 nm. There may be insufficient ascorbic acid to reduce the Au^{3+} ions to Au^+ and then to Au^o atoms when the seeds were available in the reaction mixture. The star-like morphologies of gold nanoparticles were produced when the reduced Au^o atoms tended to adsorb on pre-existing NPs facets [2]. Furthermore, the red-shift of the SPR to the longer

 This journal is © The Royal Society of Chemistry 20xx

Please do not adjust margins

Please do not adjust margins

wavelength could attribute to the formation of larger size of AuNSs or the ratio of core size to the branches length [3]. To assess the impact of ascorbic acid on the morphologies of AuNSs-FS, SEM analysis was conducted on colloid samples prepared with 30, 40, and 50 µL of 0.1 M AA. The results showed that with low volumes of ascorbic acid (30 µL), the nanoparticles took on spherical or pseudo-spherical shapes or had short branches protruding from the core. (Fig. S3-B). Continue to increase the volumes of the ascorbic acid to 40 µL, the branches formed with long length and sharp, the number of branches grew more, and the average number of branches per single particle could be more than 15 (Fig S3-C). However, for the colloid samples prepared at a volume above 40 µL, the core size seems more extensive, and the branches' lengths are shorter than the particles synthesized at the low volume of ascorbic acid. It might be due to using a large amount of ascorbic acid; more Au^{o} atoms were formed based on absorption on the un-binding Ag^{o} crystalline facets. These reduced Au^{o} atoms were continuously concentrated on these facets, thus that make the AuNSs core grow bigger (Fig S3-D). By investigating the impact of ascorbic acid on the development of AuNSs-FS, we can better understand the growth mechanism of nanoparticles, which provides more information in the controlled conditions to obtain well-defined star-like gold nanoparticles.

Effect of the $HAuCl_4$ precursors

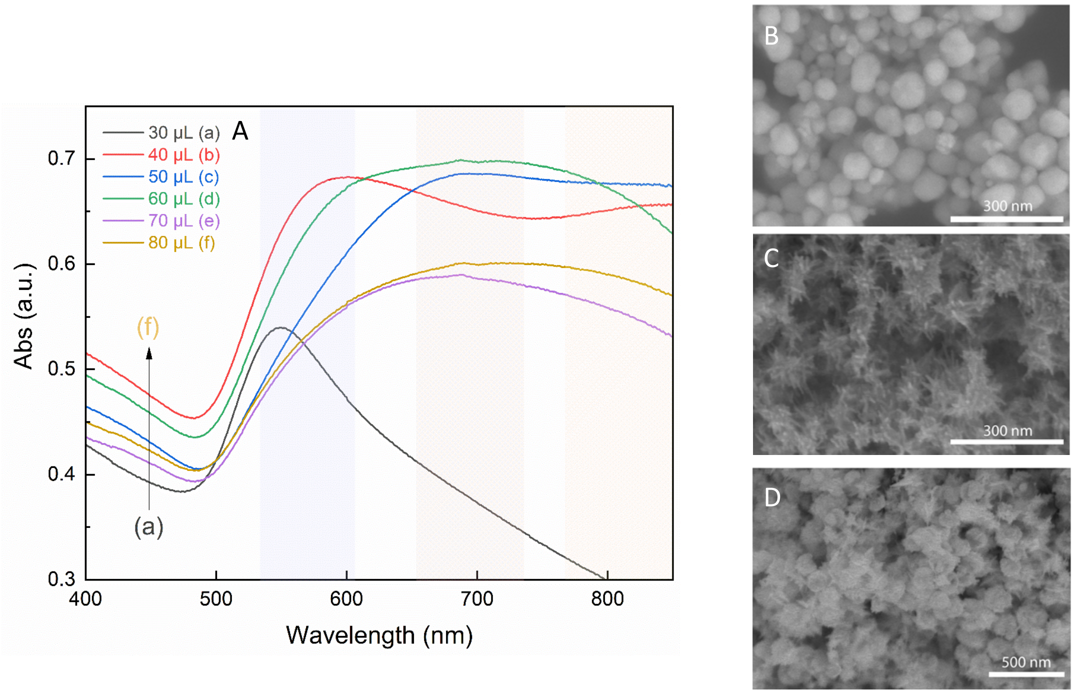

Fig. S3. (A) UV-Vis spectrum depicting the effect of different ascorbic acid volumes ranging from 30 to 80 µL (a-f curves) on the formation of star-liked gold nanoparticles. SEM images of the AuNSs-FS synthesized with (B) 30, (C) 40, and (D) 50 µL of ascorbic acid (0.1 M).

Please do not adjust margins

Please do not adjust margins

A series of samples with various $HAuCl_4$ precursor volumes ranging from 7.0 to 15 mL was prepared to study the optical properties and morphologies of the gold nanostars. Based on the results depicted in Fig. S4, when the volume of $HAuCl_4$ was increased to 10 mL, the spectrum exhibited a high intensity, with the maximum absorbance peak appearing at around 745 nm. However, upon further increasing the volume of $HAuCl_4$ beyond 10 mL, the absorbance intensity decreased remarkably from 0.85 to 0.74. Additionally, a red shift towards longer wavelengths was observed, and no absorbance peak was detected on the UV-Vis spectrum. That can be explained due to the use of two more precursors in the reaction system could cause more Au^o atoms to be generated from the reaction system. These atoms tend to grow in different shapes besides the gold nanostars. Additionally, producing more atoms also led to the unexpected adsorption of these atoms on the pre-formed seeds, forming the bigger size of Au nanoparticles. That could affect the optical properties of AuNSs [4]. Moreover, the morphologies of the nanoparticles obtained were analyzed using SEM microscopy. At 10 mL of 0.25 mM $HAuCl_4$, the particles formed numerous protrusion branches that were long, sharp, and thin, having a high aspect ratio. (Fig S4-B). For the sample prepared with 15 mL of 0.25 mM $HAuCl_4$, the nanoparticles were formed with many nanometer-sized tips with different branch lengths and core diameters (Fig S4-C). These results are consistent with the prediction in the UV-Vis spectrum based on the broad absorption that occurs in the near-infrared regions.

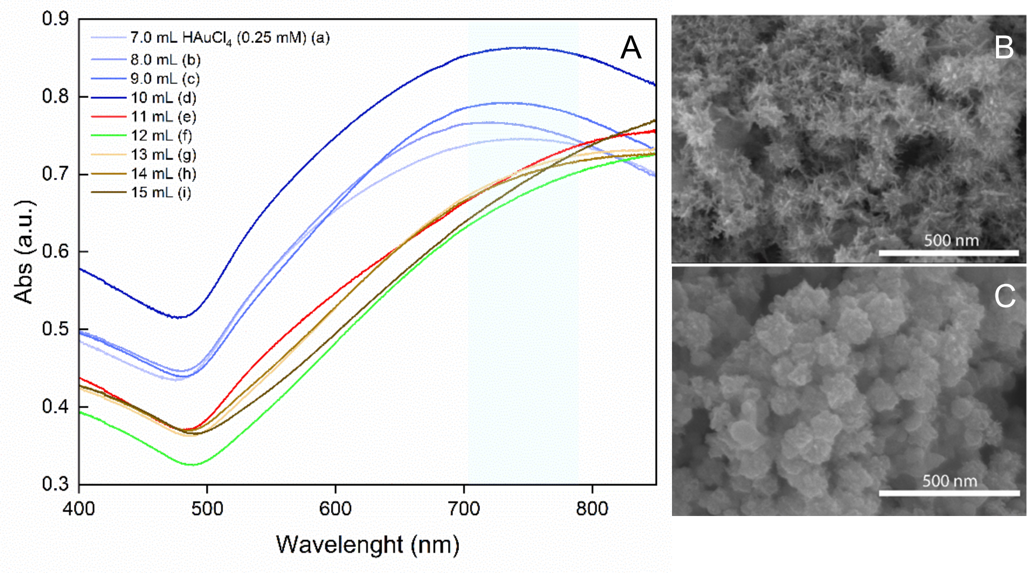

Fig S4. (A) UV-Vis spectra of the colloidal samples synthesized with difference volumes of precursor $HAuCl_4$ 0.25 mM ranging from 7.0 to 15 mL $HAuCl_4$ (0.25 mM), (B) and (C) SEM images of the obtained AuNSs-FS prepared with 10 and 15 mL of $HAuCl_4$

 This journal is © The Royal Society of Chemistry 20xx

Please do not adjust margins

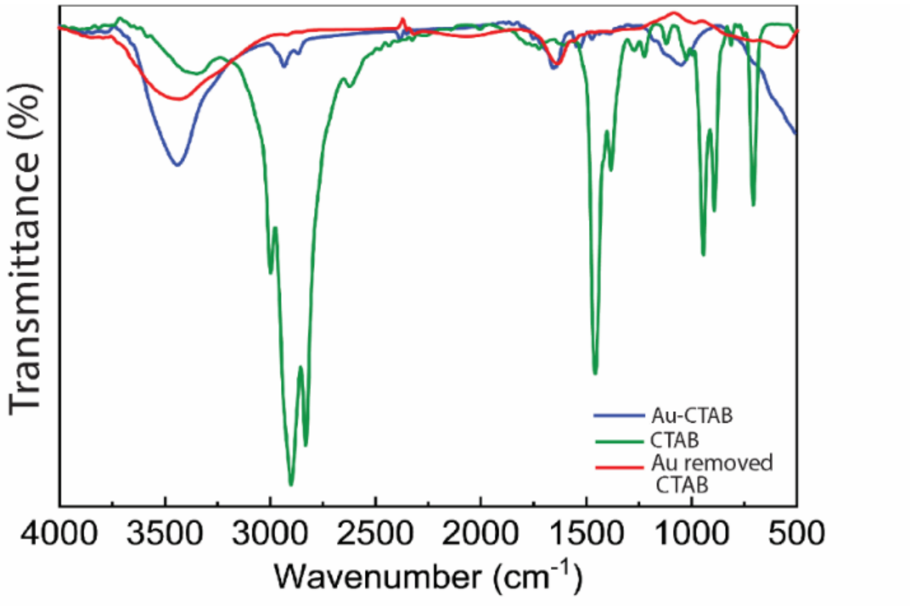

Fig S5. FTIR spectrum of the (A) AuNSs-CTAB, (B) pristine CTAB, and (C) the AuNSs after removing unadsorbed CTAB from the solution and a part of CTAB on the surface of the nanoparticle.

FTIR of the AuNSs-CTAB before and after rinsing with HCl

The CTAB removal process with HCl was evaluated with FTIR spectroscopy. The characteristic peaks observed at 2919.7 and 2850.3 cm^{-1} in the spectrum of CTAB and AuNS-CTAB are assigned to the asymmetric and symmetric stretching vibration of C-CH_2 in the methylene chains, respectively [5]. After treatment with 5.0 mM HCl aqueous solution, these peaks disappeared in the spectrum of AuNR-CTAB (Fig. S5). It could be inferred that a part of the CTAB was removed from the surface of AuNSs. The removal of CTAB allows AuNSs to have the enlarged surface areas directly exposed to the surrounding environment, making it easier to cover with the silver layer. Additionally, partial removing CTAB from the surface could limit the noise signal caused by the CTAB molecules when determined the SERS signal of the analyte molecules.

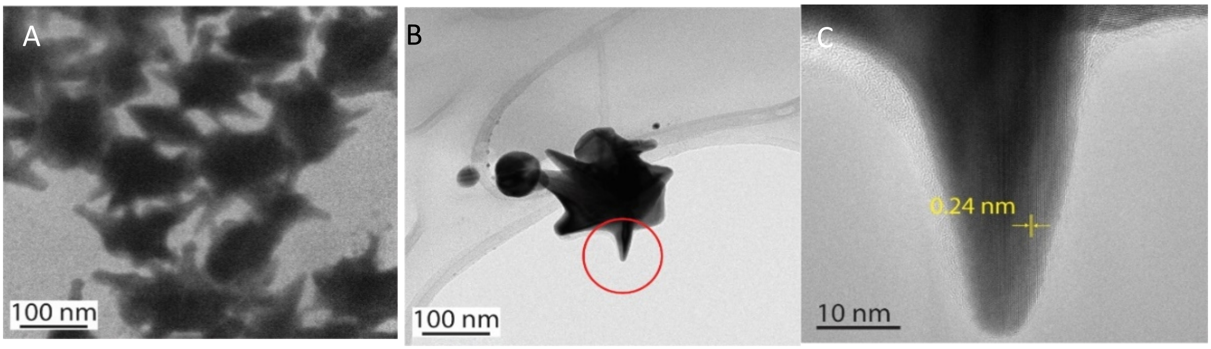

Fig. S6 (A) TEM images of AuNSs synthesized at 0.8 mL of seed solution and 10 mL of growth solution, (B) a typical AuNS particle prepared with CTAB at the same condition after removing unadsorbed CTAB through the rinsing process, there is no significant change in the AuNSs shape before and after rinsing, and (C) A relative high-resolution TEM micrograph taken on a selected area of the AuNSs spike, showing the interplanar spacing of 0.24 nm associated with (111) planes in facet-centered cubic.

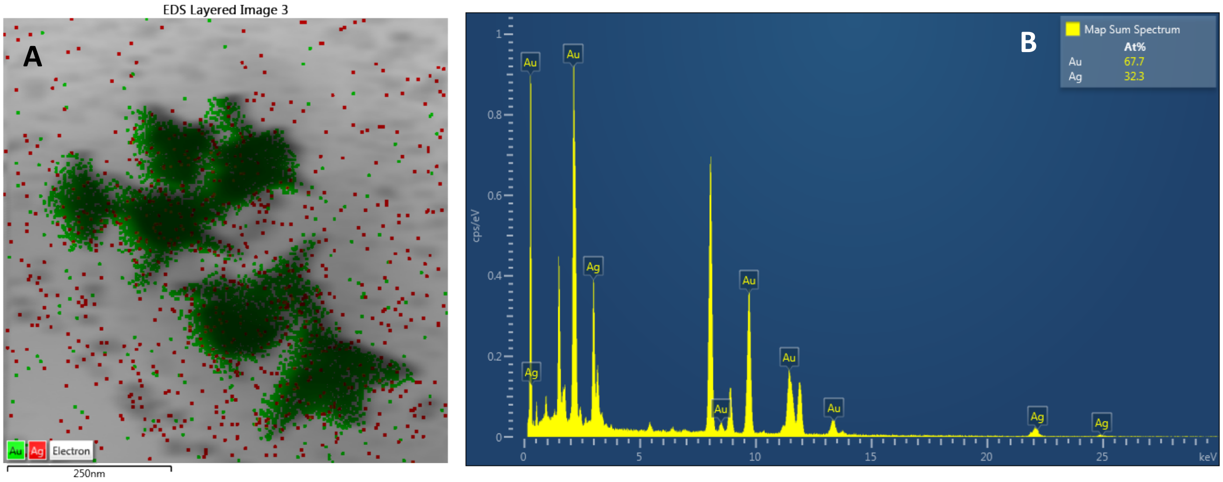

Fig. S7 (A) Overlay EDS signal mapping of Au and Ag elements of the AuNSs@Ag sample synthesized at 200 µL of 10 mM $AgNO_3$, (B) corresponding EDS map summary spectrum shows the atom percentage of Au and Ag.

z

This journal is © The Royal Society of Chemistry 20xx

Please do not adjust margins

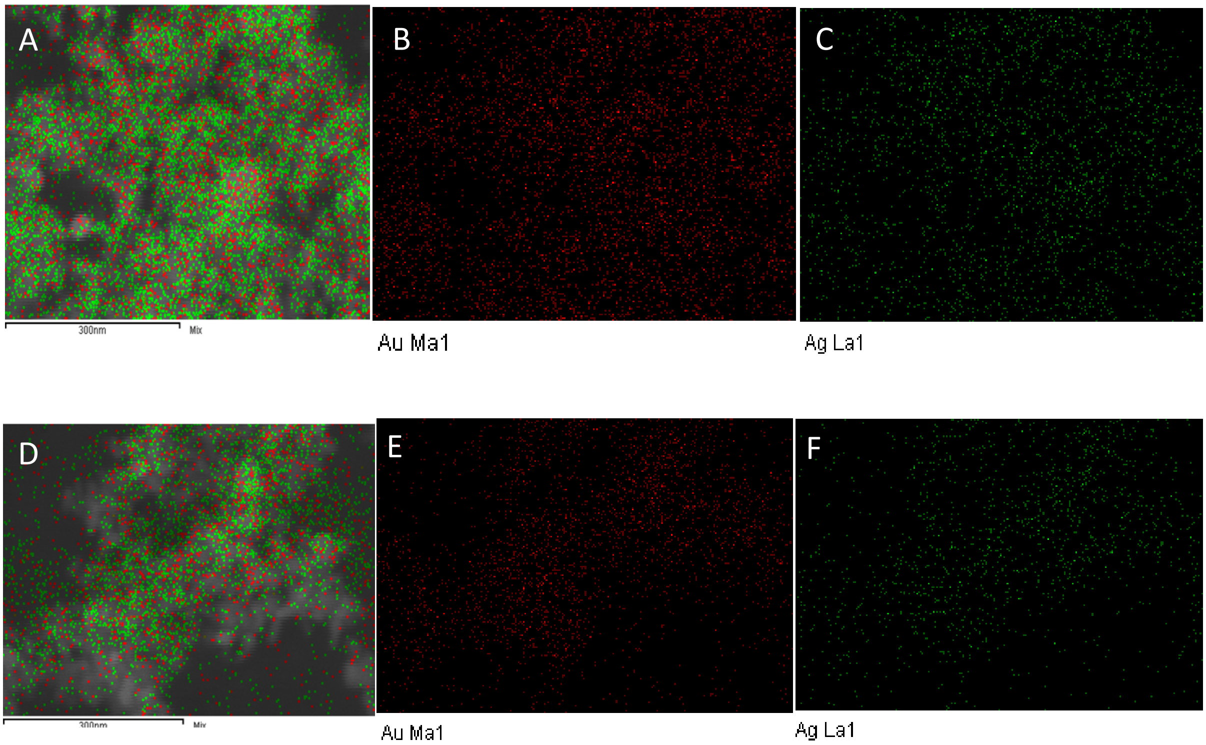

Fig. S8 EDS elemental mapping analysis for the (A) Overlay AuNSs-FS@Ag, (B) Au, (C) Ag distribution of the self-asembled nanoarrays. And, (D-F) the elemental mapping of overlay AuNSs-CTAB@Ag, Au, and Ag distribution in the nanoarrays, respectively.

This journal is © The Royal Society of Chemistry 20xx

Please do not adjust margins

Determination of the enhancement factor (EF)

For calculation the enhancement factor EF contributed from the AuNSs@Ag-FS, we used the following Eq. 1:

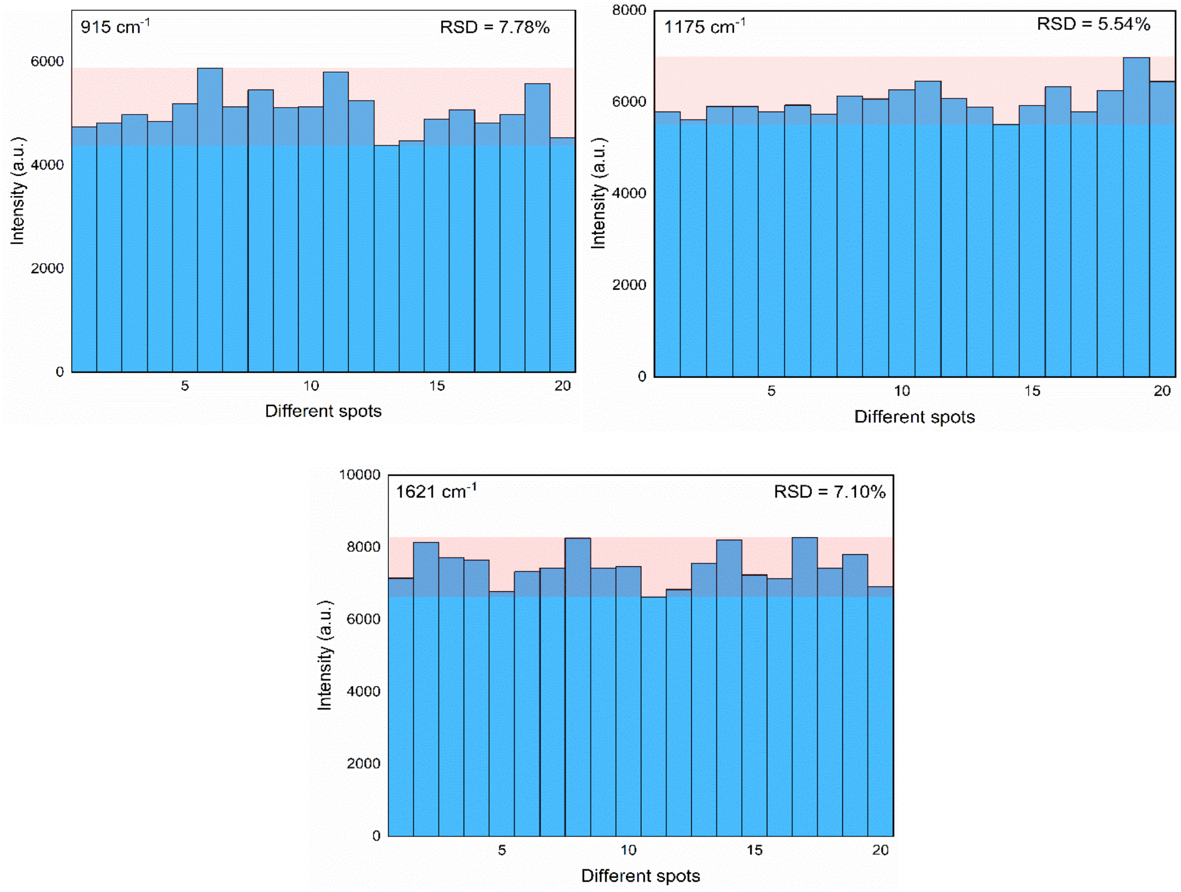

Fig. S9 The SERS intensities of CV at (A) 915 cm^{-1} , (B) 1175 cm^{-1}, and (C) 1621 cm^{-1} from 20 random detection spots from AuNSs@Ag-CTAB nano-substrates and corresponding RSD values.

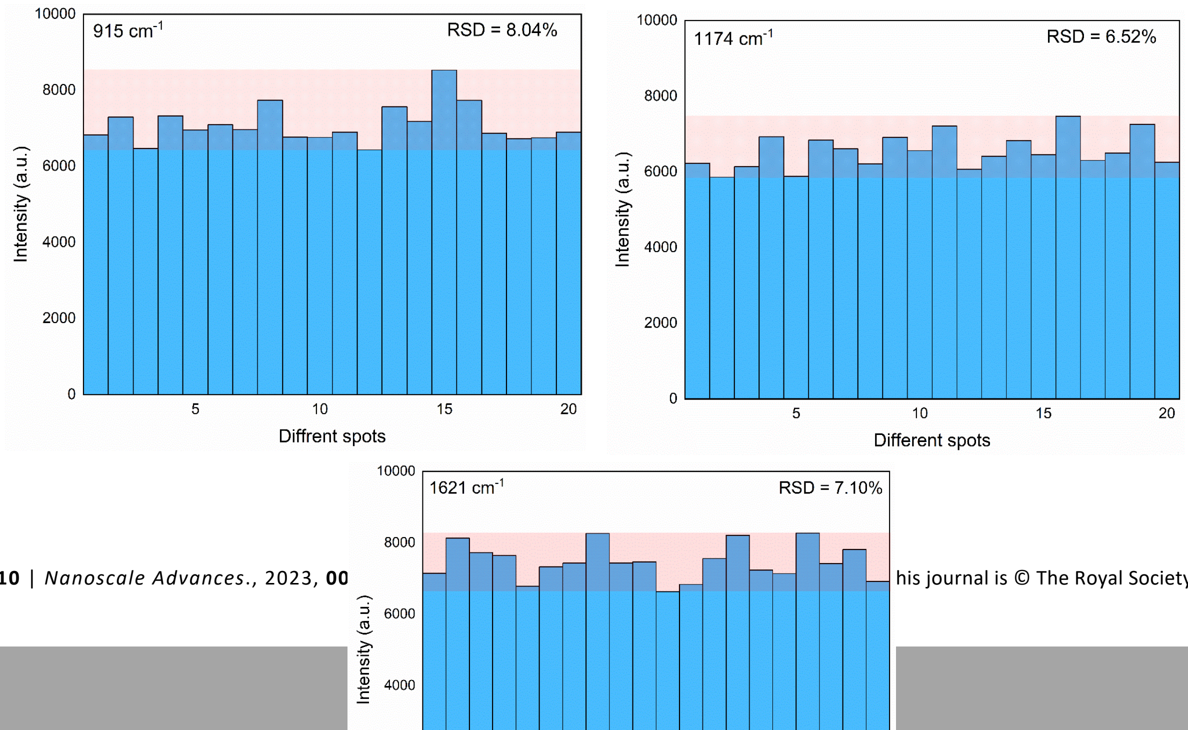

his journal is © The Royal Society of Chemistry 20xx

$$EF = \frac{I_{SERS}}{I_{Raman}} \times \frac{N_{Raman}}{N_{SERS}}$$

N_{Raman} = (area of excited laser/Raman area) x ($N_{avogadro}$ x $V_{CV/silicon\ wafer}$ x C_M of CV)

N_{SERS} = (area of excited laser/SERS area) x ($N_{avogadro}$ x $V_{CV/nanosubstrates}$ x C_M of CV on nano-substrate).

The SERS substrate area = 0.36 cm^2.

Raman area = 1.5 x SERS area

The volume of CV on the silicon wafer = the volume of CV on the nano-substrate.

$C_{M\ CV/silicon\ wafer}$ = 5 x 10^5 x $C_{M\ of\ CV/AuNSs@Ag-FS}$.

I is intensity of the 1372 cm^{-1} peak with the I_{SERS} of 8984 a.u. and I_{Raman} of 246 a.u.

$$\frac{I_{SERS}}{I_{Raman}} = 36.52$$

$$\frac{N_{Raman}}{N_{SERS}} = 0.66 \times 5 \times 10^5$$

$$EF = \frac{I_{SERS}}{I_{Raman}} \times \frac{N_{Raman}}{N_{SERS}}$$ = 1.2 x10^7 for the AuNSs@Ag-FS nano-substrate.

For the AuNSs@Ag-CTAB, by using this calculation method, the EF value was determined at 3.43 x 10^5.

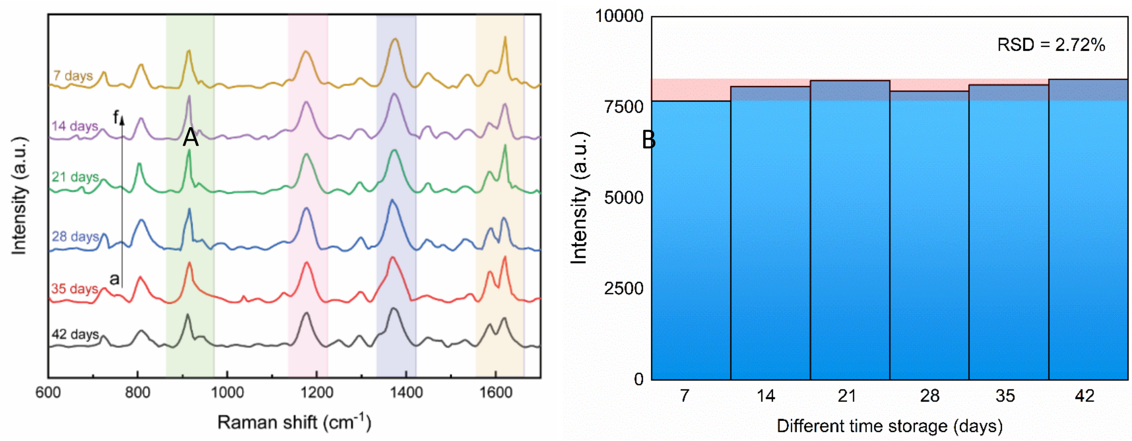

Fig. S11 (A) SERS experiments on the stability of self-assembled AuNSs-FS@Ag nanoarrays stored after 7, 14, 21, 28, 35, and 42 days and studied on the 2.0 ng/mL of CV aqueous solution. (B) The SERS intensities of the peak at 1372 cm^{-1} were investigated on various AuNSs-FS@Ag nanoarray substrates after 42 days of storage.

Table S1. XPS spectrum data with the binding energy (eV) peaks positions for the AuNSs-FS, AuNFs-FS@Ag, AgNPs samples.

**Nanoparticles**	**Au $4f_{5/2}$**	**Au $4f_{7/2}$**	**Ag $3d_{3/2}$**	**Ag $3d_{5/2}$**	**O 1s**	**C 1s**

Please do not adjust margins

AuNSs-FS	87.58	83.88	-	-	532.28	284.38
AgNPs	-	-	373.98	367.98	532.18	284.48
AuNSs-FS@Ag	87.68	83.98	374.08	368.08	532.28	284.88

eV, electron volts.

Table S2. Linear correlation between different concentrations of CV and the intensity of SERS at specific peaks.

Raman shift (cm^{-1})	Linear equation	R^2 value
**AuNSs-FS@Ag self-assembled nanoarrays**		
915	y = 2096758 x + 2003.6	0.963
1174	y = 2426690 x + 1188.3	0.982
1372	y = 2340970 x + 1846.8	0.995
1621	y = 2846553 x + 1034.9	0.973
**AuNSs-FS@Ag self-assembled nanoarrays**		
915	y = 27467 x + 532	0.982
1174	y = 27824 x + 708	0.984
1372	y = 29103 x + 651	0.997
1621	y = 30544 x + 1034	0.982

2. Experimental Section

Removing process of the surfactant from the as-prepared AuNSs-CTAB

The unadsorbed CTAB surfactants remained in the colloidal solution, and part of the CTAB physisorbed on the AuNSs surface was washed using a modified version of Wang et al.'s method [6] for further coverage with the Ag shell. First, a solution of HCl with either 5 mM concentration was added to the dispersion, lowering the pH to 2.0. The mixture was then stirred at room temperature for 4 hours. The resulting colloid was centrifuged twice at 10,000 rpm for 10 minutes to remove excess surfactant. The sedimentation fractions were then redispersed in Mili-Q water for subsequent analysis.

References

1. H. A. Day, D. Bartczak, N. Fairbairn, E. McGuire, M. Ardakani, A. E. Porter and A. G. Kanaras, CrystEngComm, 2010, 12, 4312-4316.
2. W. Wang, Y. Pang, J. Yan, G. Wang, H. Suo, C. Zhao and S. Xing, Gold Bulletin, 2012, 45, 91-98.

 This journal is © The Royal Society of Chemistry 20xx

Please do not adjust margins

Please do not adjust margins

3. F. Liebig, R. Henning, R. M. Sarhan, C. Prietzel, C. N. Z. Schmitt, M. Bargheer and J. Koetz, RSC Advances, 2019, 9, 23633-23641.
4. Y. Zhang, B. Wang, S. Yang, L. Li and L. Guo, New Journal of Chemistry, 2015, 39, 2551-2556.
5. G. Su, C. Yang and J.-J. Zhu, Langmuir : the ACS journal of surfaces and colloids, 2015, 31, 817-823.
6. Z. Wang, S. Zong, J. Yang, C. Song, J. Li and Y. Cui, Biosensors & bioelectronics, 2010, 26, 241-247.

This journal is © The Royal Society of Chemistry 20xx

Please do not adjust margins
